# Raman‐activated cell sorting and metagenomic sequencing revealing carbon‐fixing bacteria in the ocean

**DOI:** 10.1111/1462-2920.14268

**Published:** 2018-07-02

**Authors:** Xiaoyan Jing, Honglei Gou, Yanhai Gong, Xiaolu Su, La Xu, Yuetong Ji, Yizhi Song, Ian P. Thompson, Jian Xu, Wei E. Huang

**Affiliations:** ^1^ Single‐Cell Center, CAS Key Laboratory of Biofuels and Shandong Key Laboratory of Energy Genetics Qingdao Institute of BioEnergy and Bioprocess Technology, Chinese Academy of Sciences Qingdao Shandong People's Republic of China; ^2^ Department of Engineering Science University of Oxford, Parks Road Oxford OX1 3PJ UK; ^3^ University of Chinese Academy of Sciences Beijing People's Republic of China; ^4^ Disease and Fishery Drugs Research Center Marine Biology Institute of Shandong Province Qingdao Shandong People's Republic of China

## Abstract

It is of great significance to understand CO_2_ fixation in the oceans. Using single cell Raman spectra (SCRS) as biochemical profiles, Raman activated cell ejection (RACE) was able to link phenotypes and genotypes of cells. Here, we show that mini‐metagenomic sequences from RACE can be used as a reference to reconstruct nearly complete genomes of key functional bacteria by binning shotgun metagenomic sequencing data. By applying this approach to ^13^C bicarbonate spiked seawater from euphotic zone of the Yellow Sea of China, the dominant bacteria *Synechococcus* spp. and *Pelagibacter* spp. were revealed and both of them contain carotenoid and were able to incorporate ^13^C into the cells at the same time. Genetic analysis of the reconstructed genomes suggests that both *Synechococcus* spp. and *Pelagibacter* spp. contained all genes necessary for carotenoid synthesis, light energy harvesting and CO_2_ fixation. Interestingly, the reconstructed genome indicates that *Pelagibacter* spp. harbored intact sets of genes for β‐carotene (precursor of retional), proteorhodopsin synthesis and anaplerotic CO_2_ fixation. This novel approach shines light on the role of marine ‘microbial dark matter’ in global carbon cycling, by linking yet‐to‐be‐cultured *Synechococcus* spp. and *Pelagibacter* spp. to carbon fixation and flow activities *in situ*.

## Introduction

Microorganisms in the oceans are responsible for approximately half of the carbon dioxide fixation on Earth (Sabine *et al*., 2004), yet we know little about their ecological and functional roles *in situ* (Amann *et al*., 1995; Whitman *et al*., 1998; Dumont and Murrell, 2005). It is hypothesized that significant populations of uncultivated microorganisms play a pivotal role in carbon fixation in oceans. As it is a challenge to establish a direct link between the phenotype (functions) and the genotype (genomics) of cells within a complex microbial community, the actual identity and activity of these contributors in the oceans remain elusive (Weller *et al*., 1992; Zehr *et al*., 2008; Tripp *et al*., 2010).

This hurdle is mainly caused by the dominant and not‐yet‐cultured marine microbes (Venter *et al*., 2004). More importantly, studies based on pure cultures are insufficient to reveal bacterial ecological function in their natural and biological context (Huang *et al*., 2009). As a result, many ecologically important but uncultured microbes were first discovered by advanced molecular tools, in the absence of direct validation of *in situ* functional activities. For example, the introduction of metagenomic approaches has enabled discovery of SAR11 bacteria (Pelagibacterales) as one of the most abundant bacteria in global oceans in 1990 (Giovannoni *et al*., 1990; Giovannoni, 2017), although it remained uncultured until 2002 (Rappe *et al*., 2002). In recent years, metagenomic analyses, which circumvent cultivation procedures and directly sequence DNA extracted from the environment (Handelsman, 2004), have already revealed massive amounts of diversity and novel genotypes from microbial ecosystems (Nayfach and Pollard, 2016). However, linking such genotypes (e.g., species identity and genes) to the phenotypes (e.g., biochemical profile and functions) *in situ* remains difficult. The ability to distinguish or validate ecological functions at the single cells is usually completely lost during the metagenomic sequencing process where all the cells in microbial community were extracted for DNA as a mixture, regardless of their specific functions (Huang *et al*., 2015; Nayfach and Pollard, 2016).

Single‐cell Raman spectra (SCRS) reflect phenotypic and intrinsic biochemical fingerprints of individual cells (Huang *et al*., 2004; Xu *et al*., 2017). Certain Raman bands in SCRS would shift when cells incorporate stable isotopes (e.g., ^13^C, ^15^N, and ^2^H), indicating metabolism of a specific isotopic substrate or general metabolic activity (Huang *et al*., 2004; Li *et al*., 2012b; Wang *et al*., 2016, 2013a; Berry *et al*., 2015; Tao *et al*., 2017). Raman‐stable isotope probing (Raman‐SIP) has been employed to probe bacterial function at the single cell level (Huang *et al*., 2009; Bankevich *et al*., 2012; Li *et al*., 2012b; Wang *et al*., 2016a). To link the function to its underlying genotypes, we have developed a series of Raman‐Activated Cell Sorting (RACS) techniques, such as Raman‐activated cell ejection (RACE) (Wang *et al*., 2017) and Raman‐activated microfluidic sorting (RAMS) (Zhang *et al*., 2015b; McIlvenna *et al*., 2016; Song *et al*., 2017), to isolate individual cells based on their SCRS characteristics. After RACS, genomic DNA from single cells was amplified by multiple displacement amplification (MDA) and subsequently processed for sequencing (Song *et al*., 2017).

However, genome coverage from RACS was frequently low when sequencing one cell at a time (< 20%), which was presumably due to severe non‐specific and biased amplification when starting from a very low amount of DNA template. Recently mini‐metagenomics, which starts from a small number of cells rather than one single cell, has been reported to improve the genome coverage and assembly integrity (McLean *et al*., 2013; Yao *et al*., 2017; Yu *et al*., 2017). Moreover, deep sequencing and advanced binning algorithms can facilitate informative mining from the assembled genomic sequences (Albertsen *et al*., 2013; Langille *et al*., 2013; Nielsen *et al*., 2014; Magnusdottir *et al*., 2017). We proposed that, mini‐metagenome from RACE sorted cells can serve as reference to reconstruct genomes encoding enzymes for complete metabolic pathways by recruiting and binning deep metagenomic sequencing data, thus establish a direct link between the phenotype (*in situ* functions) and genotype (genomes) of bacteria.

Nearly all photosynthetic cells contain carotenoids, which generate a strong resonance Raman signal. The Raman shifts of carotenoid bands caused by ^13^C incorporation in SCRS can indicate CO_2_‐fixing activity in photosynthetic cells (Li *et al*., 2012b). By application of RACE and subsequent DNA sequencing to marine bacteria, we reconstructed near‐complete genomes encoding metabolic pathways of two key bacteria responsible for CO_2_ fixation in the Yellow Sea of China. First, the CO_2_‐fixing bacterial cells were detected by Raman‐SIP after spiking ^13^C bicarbonate to the seawater. Second, RACE was applied to isolate bacterial cells that showed ^13^C shift in SCRS, and subsequently sequenced. Third, genes encoding metabolic pathways from the mini‐metagenomes were identified according to characterisation for both carotenoid synthesis and CO_2_‐fixing functions that were revealed by SCRS. Finally, the mini‐metagenomes were employed as reference to guide reconstruction of functional single‐cell genomes from deep metagenomic sequencing. It reveals a significant role of CO_2_ fixation of *Synechococcus* spp. and *Pelagibacter* spp. in the seawater.

## Results

### An ‘all‐in‐one’ device that integrates Raman‐activated cell sorting and sequencing

**Figure 1:**
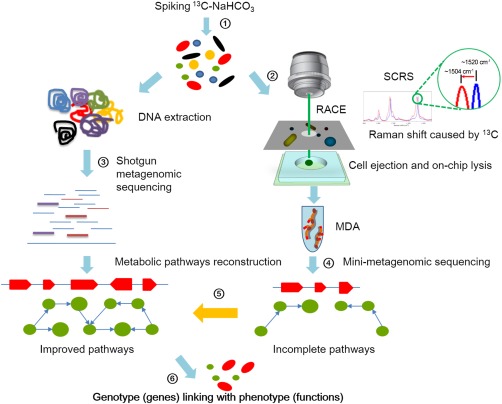
Pipeline of metagenomics aided Raman‐activated cell ejection (RACE) that links genotype to phenotype. ① Feeding of ^13^C substrate to probe the metabolism of microbial community, for example, from a seawater sample; ② cells were split into the following two processes: DNA extraction and cell sorting according to SCRS, which exhibits shifts caused by ^13^C incorporation; ③ the extracted DNA is processed for metagenomic sequencing; ④ the sorted cells are lysed and the mini‐metagenomes were amplified by MDA; ⑤ incomplete pathways from mini‐metagenomic sequencing serve as a reference of metagenomic data to reconstruct nearly complete metabolic pathways and ⑥ metabolic functions are validated by the reconstructed metabolic pathways and genes, and a link between genotype (genes) and phenotype (functions) is established at the single cell level. [Colour figure can be viewed at http://wileyonlinelibrary.com]

The coupling of Raman Activated Cell Ejection (RACE) and subsequent single‐cell sequencing is achieved via a specially designed device (Fig. 1 and Supporting Information Fig. S1). In our previous design, the slide with cell sample had to be manually flipped over for cell ejection (Song *et al*., 2017). This procedure increases the risk of contamination for single‐cell DNA amplification and slows down the sorting process and represents a hurdle for automation.

To ensure precise yet efficient workflow and prevent environmental contamination, an ‘all‐in‐one’ device was designed and fabricated, which consists of three components, assembled in a sandwich‐like manner, that couples SCRS measurement (the sampling chip), cell ejection (the transparent ejection slide), and on‐chip DNA extraction (the collection chip) in a closed format (Fig. 1 and Supporting Information Fig. S1). The process starts with adding samples onto the wells of the sampling slide (Fig. 1 and Supporting Information Fig. S1B). Then, at the SCRS measurement, the cells were not directly exposed to the Raman laser; instead, the Raman incident laser went through the quartz slide before it reached the cells. Raman signal was acquired after passing through the same quartz slide and coating layer (Supporting Information Fig. S1). As a result, the new design avoids the need to physically flip the ejection chip between SCRS acquisition and cell ejection, reducing the risk of DNA contamination and increasing the throughput of RACE (Fig. 1).

### Supplementation of ^12^C/^13^C NaHCO_3_ had a minimal effect on abundance and diversity of microbial community in seawater samples

The seawater samples were collected from a typical epipelagic (euphotic) zone in the Yellow Sea of China (Supporting Information Fig. S2; physiochemical parameters in Supporting Information Table S1). Pre‐experiment showed that the marine bacteria with ^13^C incorporation were detectable using Raman micro‐spectroscopy after 5‐day incubation with ^13^C NaHCO_3_ (Supporting Information Figs S3 and S4). To assess the influence of supplemented biocarbonate (e.g., ^12^C NaHCO_3_ and ^13^C NaHCO_3_) on microbiota structure, 16S rRNA amplicon sequencing results were compared among the original seawater (Primary), 5‐day seawater spiked with ^13^C NaHCO_3_ (13C), 5‐day seawater with ^12^C NaHCO_3_ (12C) and the 5‐day seawater control without any treatment (C_free). For each of these conditions, triplicate samples were taken, with 12 samples in total. For each sample, averaged 11.4 million 16S rDNA read pairs were sequenced (Supporting Information Table S2). After read trimming, screening and removal of chimeras and singletons, averagely ∼ 96,399 high‐quality reads per sample were obtained (Supporting Information Table S2), revealing a total of 4466 OTUs from the 12 samples.

**Figure 2:**
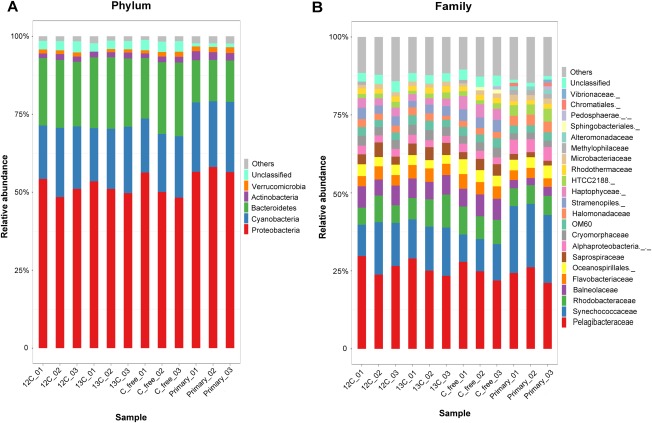
Taxonomical structure and relative abundance of each sample at the phylum level (A) and at the family level (B). ‘Primary’ represents the original seawater, ‘13C’ represents the 5‐day seawater spiked with ^13^C NaHCO_3_, ‘12C’ represents the 5‐day seawater with ^12^C‐NaHCO_3_ and ‘C_free’ represents the 5‐day seawater control without any treatment. For each of the conditions, triplicate samples were taken marked as _01, _02 and _03. Those accounting for < 1% of the total OTUs are included in ‘Others’, which are shown in gray color at the top of each bar. [Colour figure can be viewed at http://wileyonlinelibrary.com]

The rarefaction curves suggested that the sequencing depth was sufficient to capture the diversity of the microbial community (Supporting Information Table S2). Comparison of α diversity indexes (including Observed_OTUs, Simpson, Chao1 and Shannon; Supporting Information Table S2) revealed no significant difference between the four conditions in species richness and bacterial diversity (Kruskal–Wallis test or Wilcoxon test for pairwise comparison; all *p* > 0.05; Supporting Information Fig. S5). Abundance distribution at the phylum and family levels suggested that supplementation of carbon source ^12^C/^13^C NaHCO_3_ had introduced minor influence on the structure of microbial community (Fig. 2). At the phylum level, Cyanobacteria, Bacteroidetes, and Proteobacteria were the most abundant taxa, contributing to 91.58%–93.26% of the bacterial communities in each sample (Fig. 2A). Classification at the family level detected no significant difference either in microbiota structure among the four conditions of seawater (Fig. 2B). Apart from the (bacterio)chlorophyll‐based phototrophs of Synechococcaceae, Balneolaceae and Rhodobacteraceae, proteorhodopsin‐based Pelagonacteraceae (SAR11) were amongst the most dominant bacteria within the community (Fig. 2B).

### Raman shift biomarkers indicated CO_2_ fixing activities

**Figure 3:**
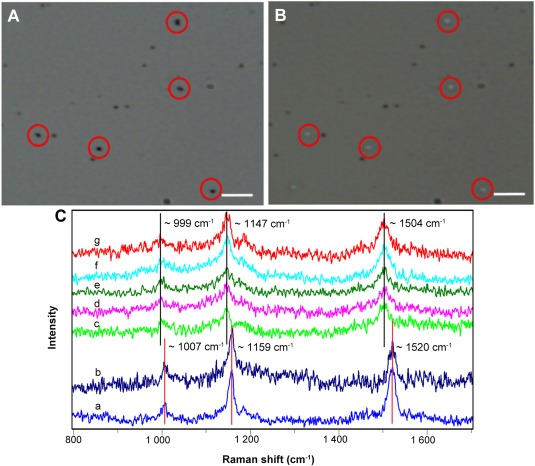
Raman‐activated cell ejection of CO_2_‐fixing microbes in the sea water sample for mini‐metagenomics. A. Cells on the ejection slide were identified by Raman measurement. B. Cells were ejected off the slide. C. Single‐cell Raman spectra of (a) cells treated with ^13^C NaHCO_3_ at time *t* = 0 days; (b) cells treated with ^12^C NaHCO_3_ at time *t* = 5 days; (c–g) five cells shown in (A) and (B) that were treated with ^13^C NaHCO_3_ at time *t* = 5 days. The scale bar in (A) and (B) is 10 µm. [Colour figure can be viewed at http://wileyonlinelibrary.com]

Single‐cell Raman spectroscopy coupled with stable‐isotope probing (SIP) has been used to reveal specific metabolic functions or to screen for the active species in a microbial community, which is based on the fact that the integration of stable isotope into a cell causes significant shifts of Raman bands in SCRS (Li *et al*., 2012b; Wang *et al*., 2016a). Carotenoid vibrations are dominant Raman signals in SCRS of photosynthetic cells, and Raman shifts on carotenoid bands indicate ^13^C incorporation into the whole cells, which provides unambiguous indication of CO_2_ fixation (Li *et al*., 2012b). The characteristic Raman spectra of carotenoid‐containing cells are in the ranges of 1500–1550, 1150–1170 and 1000–1020 cm^−1^, which are caused by in‐phase C=C (*v*1), C–C stretching (*v*2) vibrations of the polyene chain and in‐plane rocking mode of CH_3_ groups attached to the polyene chain (*v*3) (Schulz and Baranska, 2007; Robert, 2009; Li *et al*., 2012a). Although *v*1, *v*2 and *v*3 positions in SCRS of photosynthetic cells might vary, they would all shift to lower wavenumbers when cells have incorporated ^13^C (Li *et al*., 2012a). After analyzing many different photosynthetic cells with ^13^C incorporation (Li *et al*., 2012b), it is found that ^13^C in cells usually cause the *v2* and *v3* bands shifting to the wavenumbers lower than 1150 and 1000 cm^−1^ respectively. For example, after *Synechococcus* sp. PCC7002 grown in ^13^C NaHCO_3_, its *v2* and *v3* bands shift to 1147 and 999 cm^−1^ from original 1159 and 1008 cm^−1^ in SCRS of ^12^C cells (Supporting Information Fig. S3C). Hence, in this study, we used the simultaneous shifts in *v2* and *v3* as criteria, to identify and sort photosynthetic cells that are actively taking in ^13^C NaHCO_3_ (Fig. 3 and Supporting Information Fig. S3C).

### Isolation of CO_2_‐fixing cells from seawater samples based on their characteristic SCRS

^13^C shift SCRS in cells were observed in day five after continuous monitoring seawater reactors. Cells were sorted using RACE illustrated in Fig. 1 and Supporting Information Fig. S3. In the seawater samples spiked with ^13^C NaHCO_3_, cells with ^13^C shifted carotenoid bands in SCRS were sorted (Fig. 3C), based on the Raman sorting criteria (Supporting Information Fig. S3C). One group was taken as negative control with no cells (details showed in Supporting Information Fig. S6). Eight groups were sorted, each contained 30 cells, which all showed significant Raman shifts at *v*2 and *v*3 bands in SCRS, similar to those in ^13^C *Synechococcus* sp. PCC7002 (Supporting Information Fig. S3C and Fig. 3C).

To link the CO_2_‐fixing function to genotype, the sorted cells were processed for subsequent mini‐metagenomic analysis. The cell lysis was carried out on the cell collection chip (see *Experimental procedures*), and the lysate was then transferred to centrifuge tube for genomic DNA amplification. The MDA products were checked for 16S rRNA sequences by PCR to confirm successful amplification (Supporting Information Fig. S6; primers shown in Supporting Information Table S3). Among eight sorted samples, two sample groups that yield positive PCR results, which were sorted independently using identical RACE criteria from a single seawater sample, were designated as TET3 and TET4, and further processed to construct the library for high‐throughput genomic sequencing.

### Mini‐metagenomic analysis was consistent with phenotypic properties identified by RACE in terms of carotenoid synthesis and CO_2_ fixation

Due to the low rate of assembly errors (Bankevich *et al*., 2012), SPAdes was chosen to assemble the reads into contigs, with total length of 4.72 and 4.49 Mbp for TET3 and TET4, respectively (Supporting Information Table S4). For TET3 and TET4, 4.0% and 7.6% of the contigs (in length) were < 200 bp, 15.6% and 10.3% were > 1500 bp, and 80.4% and 82.1% were ranged from 200 to 1500 bp, respectively (Supporting Information Table S4). These contigs were used as draft mini‐metagenomes for downstream analysis.

**Figure 4:**
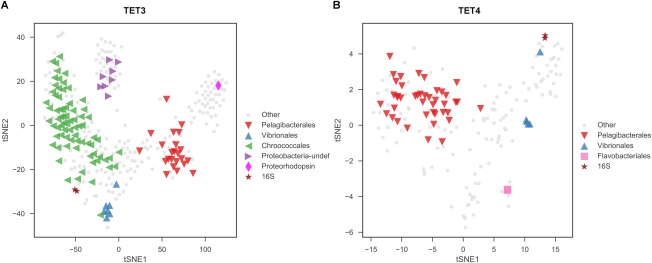
t‐SNE embeddings of the 4‐mer signatures of assembled mini‐metagenomic contigs. Plots represent samples TET3 (A) and TET4 (B), and only those contigs longer than 1000 bp were considered. Contigs were colored and shaped based on taxonomic annotations listed in Table 1. This result suggests that Pelagibacterales (including Proteobacteria‐undef) and Chroococcales were dominant in sample TET3, while Pelagibacterales was dominant in sample TET4. [Colour figure can be viewed at http://wileyonlinelibrary.com]

After decontamination, contigs from the *de novo* assembled mini‐metagenome of RACE‐sorted cells were assigned to particular taxa based on their sequence similarity (Table 1). They belonged to seven phyla: Proteobacteria, Bacteroidetes, Chlorophyta, Fusobacteria, Firmicutes, Actinobacteria and Cynaobacteria (Table 1). Contigs > 1000 bp were clustered and visualized using t‐SNE via their 4‐mer signatures, which represented the major clusters of genomes in each sample (Fig. 4 and Supporting Information Table S4). As expected, a significant portion of the contigs was not assigned to any known bacteria. On the other hand, contigs from Chroococcales (an order of Cyanobacteria) were dominant in TET3 (Fig. 4A) while those from Pelagibacterales (SAR11) were dominant in TET4 (Fig. 4B). Consistent with this, in terms of percentages of reads in mini‐metagenomic data, the most abundant bacteria were Chroococcales (an order of cyanobacteria; 66.17%) in TET3 and Pelagibacterales (SAR11, an order of Alphaproteobacteria composed of oligotrophic photochemotroph bacteria; 54.99%) in TET4 (Scanlan *et al*., 2009; Giovannoni, 2017). TET3 and TET4 shared other three phyla (Proteobacteria, Cynaobacteria and Firmicutes) and six 16S rRNA sequences in TET3 had the same taxonomy identity as TET4 (Supporting Information Table 1). Interestingly, a proteorhodopsin (PR) gene was detected in TET3 (Fig. 4A), suggesting the presence of PR‐containing bacteria in our sorted samples.

**Table 1:** Predicted genome completeness and 16S rRNA genes of RACE‐sorted bacteria.

		Metagenome	Mini‐metagenome			
RACE‐sorted samples	Class^*^	Estimated genome completeness (%)	Estimated genome completeness (%)	Identity 16S rRNA (%)	Percentage of reads^**^ (%)	References supporting presence of carotenoids
TET3	***Proteobacteria***	100	7.29		27.92	
	* Proteobacteria‐undefined*	97.41	4.17	–	2.90	Shindo and Misawa (2014)
	* Vibrionales*	50.10	3.89	99.35	0.04	Meziti et al. (2015)
	* *→ *Pelagibacterales*	100	0.17	–	16.06	Haro‐Moreno et al. (2017)
	* Xanthomonadales*	30.57	1.72	–	0.00	Paret et al. (2012)
	* Pseudomonadales*	10.42	0.00	99.36/100	0.33	Reddy and Garcia‐Pichel (2015)
	***Cyanobacteria***	100	6.55		66.17	
	* *→ *Chroococcales*	100	6.55	100	66.17	Wang et al. (2016c)
	***Actinobacteria***	100	0.86		0.01	
	* Micrococcales*	87.72	0.86	–	0.00	Trujillo (2016)
	***Firmicutes***	100	0.00		0.01	
	* Bacillales*	89.34	0.00	100	0.01	Steiger et al. (2015)
	*Undefined sequences*		–	–	5.89	–
**TE**T4	***Proteobacteria***	100	42.40		56.84	
	* *→ *Pelagibacterales*	100	13.66	–	54.99	Haro‐Moreno et al. (2017)
	* Vibrionales*	50.10	4.75	99.67/100	0.01	Meziti et al. (2015)
	* Pseudomonadales*	10.42	2.98	98.61/96.31	0.48	Reddy and Garcia‐Pichel (2015)
	* Xanthomonadales*	30.57	0.16	–	0.00	Paret et al. (2012)
	* Caulobacterales*	9.98	0.16	–	0.00	Fukui et al. (2013)
	***Bacteroidetes***	100	1.88	–	9.87	
	* Flavobacteriales*	100	1.88	–	9.87	Shahina et al. (2013)
	***Chlorophyta (Eukarya)***	77.59	0.94	–	0.00	
	* Sphaeropleales*	1.33	0.94	–	0.00	Lee et al. (2016)
	***Fusobacteria***	11.05	0.51	–	0.01	
	* Fusobacteriales*	11.68	0.51	–	0.01	–
	***Firmicutes***	100	0.00	–	4.66	
	* Bacillales*	89.34	0.00	100	0.83	Steiger et al. (2015)
	***Cyanobacteria***	100	0.00	–	0.02	
	* *→ *Chroococcales*	100	0.00	100/100	0.02	Wang et al. (2016c)
	***Undefined sequences***		–	–	28.60	–

*In this column, phylum names are in italics bold, and order names are only in italics; ‘→’ represent the bacteria that are numerically abundant, that is, the focus of this study.

**This column lists the fraction of reads mapped to the specific organisms among the reads mapped to all the annotated contigs.

RACE sorted cells should contain carotenoids and utilize ^13^C NaHCO_3_. According to literature, all bacteria identified in TET3 and TET4 were potentially able to synthesize carotenoids (Table 1), which was in good agreement with the sorting criteria. Two dominant types of cells in TET3 and TET4 were Cyanobacteria and Pelagibacterales (SAR11), both of which are able to use sunlight as energy source, although cyanobacteria use chlorophyll whilst Pelagibacterales (SAR11) employ PR to harvest sunlight energy (Finkel *et al*., 2013). It is expected that cyanobacteria were detected, as they are dominant photoautotrophic bacteria in the ocean, which usually contain carotenoids and use ^13^C NaHCO_3_. Interestingly, the sequencing data show that Pelagibacterales (SAR11) was found in TET3 (16.06%) and TET4 (54.99%), which should be ^13^C‐labelled and contain carotenoids.

### Mini‐metagenome from RACE and metagenome from whole community DNA are coupled to reconstruct genomes of dominant CO_2_‐fixing bacteria in the seawater

The 16S rRNA metagenomic sequencing also indicated that *Synechococcus* spp. (Cyanobacteria) and *Pelagibacter* spp. (Pelagibacterales) were the two dominant bacteria in the seawater (Supporting Information Fig. S7). To reconstruct their genomes, deep metagenomic sequencing of the seawater sample was performed. Over 117 million paired end clean reads were produced, which were assembled into 1,990,773 contigs (totally 1.67 Gb; Supporting Information Table S4). Among them, totally 47,274 contigs were annotated as either *Synechococcus* spp. (24,834 contigs, total length of 36.2 Mb, average GC content of 60%) or *Pelagibacter* spp. (Candidatus Pelagibacter and Pelagibacteraceae‐undef; 22,440 contigs, combined length of 20.5 Mb, average GC content of 31%). GC distribution plots of the re‐mapped sequences were smooth (Supporting Information Fig. S8), which was consistent with the absence of significant contamination. The overall GC contents of the binned genomes (represented by curve's central peak; Supporting Information Fig. S8) were close to those of known sequenced genomes of corresponding taxa, respectively (Candidatus Pelagibacter ubique HTCC1062 with RefSeq accession NC_007205.1: GC content 29.7%; *Synechococcus* spp. WH 7803 with RefSeq accession NC_009481.1: GC content 60.2%). Thus, these two sets of contigs should represent, to a certain degree, the collective genetic complements of CO_2_‐fixing *Synechococcus* spp. and *Pelagibacter* spp., respectively, in this particular ecosystem of seawater. In fact, if taken as a whole, respectively, each of them made nearly complete genomes (100% completeness; as estimated by CheckM) of *Synechococcus* spp. (Cyanobacteria) and *Pelagibacter* spp. (Pelagibacterales) (Table 1).

The seawater was sampled from the pelagic zone and had sufficient sunlight but low organic matter (chemical oxygen demand was 1.05 mg L^−1^; Supporting Information Table S1). These conditions should have favored bacteria, such as *Synechococcus* spp. (Cyanobacteria) and *Pelagibacter* spp. (Pelagibacterales), which are able to harvest sunlight energy and undertake CO_2_ fixation. Application of RACE successfully linked bacterial ecological function (e.g., CO_2_ fixation in this case) and mini‐metagenomes, which were then served as references or blueprints to bin the complete genomes of key functional bacteria *Synechococcus* spp. (Cyanobacteria) and *Pelagibacter* spp. (Pelagibacterales) with aid of high‐throughput metagenomic sequencing. As a result, RACE‐mediated single‐cell genome is effective at establishing a link between phenotype/function and genotype.

### Reconstructed metabolic pathways reveal new ecological functions of RACE‐sorted bacteria

Contigs assembled from mini‐metagenomic data with length longer than 200 bp were collected to predict functional genes in this study (Supporting Information Table S4). The number of predicted genes in these functional mini‐metagenomic samples was 4098 and 3102 for TET3 and TET4 respectively. Other details, such as the predicted CDS numbers and tRNA numbers, are provided in Supporting Information Table S5.

RACE was employed to link bacterial CO_2_ fixing function and genotype in terms of mini‐metagenome. Although the mini‐metagenome was incomplete (Table 1), it provides sufficient genetic information to reconstruct complete metabolic pathways from high‐throughput and deep metagenomic data (see *Experimental procedures*). The functional genes derived from mini‐metagenome were highly consistent with the functional genes from shotgun metagenomic data in terms of gene similarities (Supporting Information File S1 and Supporting Information Table S7). To unravel the metabolic potential of these functional mini‐metagenomes, the two bacterial mini‐metagenomic gene sets were mapped to the KEGG pathways, which predicted gene function largely based on sequence homology (Kanehisa *et al*., 2016). Within these gene sets, eight metabolic pathways were examined, including carbon metabolism (ko01200), carbon fixation in photosynthetic organisms (ko00710) and in prokaryotes (ko00720), photosynthesis (ko00195), antenna protein synthesis (ko00196), porphyrin and chlorophyll metabolism (ko00860), carotenoid (ko00906) and terpenoid biosynthesis (ko00900), which were all detected in TET3 and TET4 samples (Supporting Information Table S6 and Supporting Information File S1). A total of 371 matches in the annotation output of these two samples showed high homology to the photosynthetic carbon‐fixing genes.

**Figure 5:**
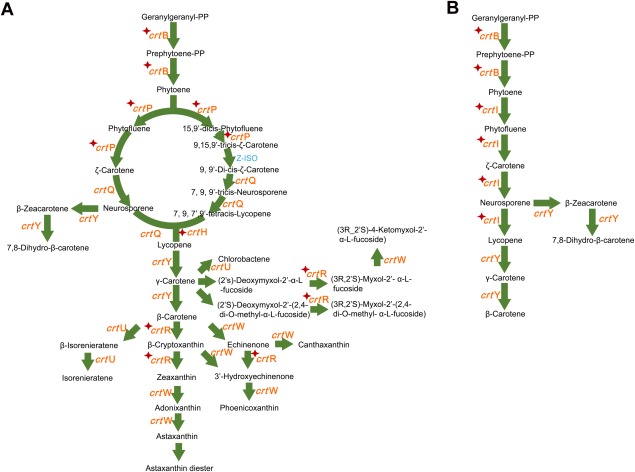
Reconstructed β‐carotene module in the carotenoid synthesis pathway by metagenomics‐aided RACE in Yellow Sea. A. Reconstructed beta‐carotene module in *Synechococcus* spp. B. Reconstructed beta‐carotene module in *Pelagibacter* spp. The known pathways were obtained from the KEGG database. Enzymes in red colour: found in mini‐metagenome guided shotgun data; with star markers: found in mini‐metagenomic data; in blue colour: not found in our data. [Colour figure can be viewed at http://wileyonlinelibrary.com]

**Figure 6:**
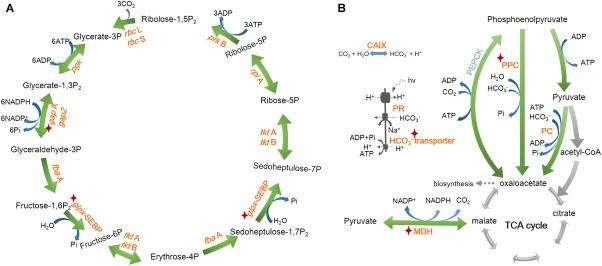
Reconstructed CO_2_ fixing pathways by metagenomics‐aided RACE in Yellow Sea A. *Synechococcus* spp. employed Calvin cycle. B. *Pelagibacter* spp. used anaplerotic reactions. Enzymes in red colour: found in mini‐metagenome guided shotgun data; enzymes with star markers: found in mini‐metagenomic data; in blue colour: not found in our data. CAIX: carbonic anhydrase; PR: proteorhodopsin; MDH: malate dehydrogenase; PPC: phosphoenolpyruvate carboxylase; PC: pyruvate carboxylase; PEPCK: phosphoenolpyruvate carboxykinase. [Colour figure can be viewed at http://wileyonlinelibrary.com]

According to Raman profiles, the sorted cells should contain carotenoids and fix CO_2_. Using mini‐metagenome as blueprints, complete pathways for carotenoid synthesis were identified for *Synechococcus* spp. and *Pelagibacter* spp. (Fig. 5). Calvin cycle for CO_2_ fixation pathways in *Synechococcus* spp. (Cyanobacteria; Fig. 6A) and anaplerotic CO_2_ fixation in *Pelagibacter* spp. (Pelagibacterales; Fig. 6B) were also revealed by the mini‐metagenomes and metagenomic bins. These observations suggested that *Synechococcus* spp. should use Calvin cycle, whilst *Pelagibacter* spp. (Pelagibacterales) may employ anaplerotic reactions to fix CO_2_ (Fig. 6 and Supporting Information File S2).

The reconstructed pathway reveals that *Synechococcus* spp. has all genes necessary for the synthesis of various carotenoids, whilst *Pelagibacter* spp. has one intact pathway for β‐carotene synthesis (Fig. 5 and Supporting Information File S2). It confirms our hypothesis that PR containing bacteria should be able to synthesize β‐carotene which can be cleaved by dioxygenase to produce retinal – an essential compound in functional PR (Von Lintig and Vogt, 2000). The presence of β‐carotene would enable *Pelagibacter* spp. self‐sufficient for PR synthesis. It also explains that carotenoid‐containing cells sorted by RACE included *Pelagibacter* spp.

In terms of sunlight harvesting system, *Synechococcus* spp. has all the genes necessary for photosynthesis, chlorophyll α, antenna (phycobilisome), Photosystem I and Photosystem II (Mulkidjanian *et al*., 2006) biosynthesis (Supporting Information Figs S9–S15 and Supporting Information File S2), which were all absent from the reconstructed genome of *Pelagibacter* spp. According to the identified proteorhodopsin (PR) genes from metagenomic bins of *Pelagibacter* spp. (Supporting Information File S3), we have successfully expressed these PR genes in *Escherichia coli* (data not shown), confirming that the sequences indeed were encoded with PR. F‐type ATPase genes were found in both *Synechococcus* spp. and *Pelagibacter* spp. (Supporting Information Fig. S12). These were expected, as *Synechococcus* spp. uses chlorophyll whilst *Pelagibacter* spp. employs PR to harvest light energy. Indeed, it has been previously reported that a pure PR‐containing strain *Dokdonia* sp. MED134, in the presence of the light, was able to fix CO_2_ using anaplerotic reactions (Palovaara *et al*., 2014). Our observation suggests that CO_2_ fixation via anaplerotic reactions by PR‐containing bacteria could happen in natural environment as well.

The above results from genome reconstruction of RACE‐sorted cells, i.e., two dominant carotenoid‐containing bacteria in the seawater *Synechococcus* spp. and *Pelagibacter* spp. exhibit active carbon fixing activities, are in good agreements with the phenotypic analysis from SCRS, which suggested that these cells were able to incorporate ^13^C from^13^C NaHCO_3_ and contain carotenoids. Reconstructed genome and pathway analysis were successful at identifying the genes responsible for carotenoid synthesis, light harvesting pathways and CO_2_ fixation pathway (Figs 5 and 6 and Supporting Information File S2). The phenotype–genotype agreement confirms that RACE sorting was effective at isolating phototrophic CO_2_ fixing bacteria in seawater and revealed a suspected yet previously unsubstantiated ecological function of the SAR11 group bacteria – *Pelagibacter* spp.

## Discussion

### RACE established a link between phenotype and genotype of cells

The composition and function of a microbial community are the two most fundamental topics in microbial ecology. Metagenomic sequencing and binning is an increasingly powerful tool which is able to give a blueprint on genotype level for profiling the microbiota directly (Albertsen *et al*., 2013; Langille *et al*., 2013; Nielsen *et al*., 2014; Magnusdottir *et al*., 2017). However, the phenotypes of the composing microbes were missed inevitably. Raman spectroscopy has been proved to be a useful approach which could provide comprehensive phenotype information of single cells in a non‐destructive manner. More importantly, by combining with the stable isotope probing method which is using stable isotope substituted substrates for keeping normal metabolism of cells, Raman‐SIP provides a promising tool for pinpointing specific metabolic functions of certain species within a complex community (Huang *et al*., 2004, 2009; Li *et al*., 2012b; Wang *et al*., 2013; Zhang *et al*., 2015a; Song *et al*., 2017) or general metabolic activity (Berry *et al*., 2015; Wang *et al*., 2016b; Tao *et al*., 2017) at single‐cell level. Given above merits, Raman activated cell sorting and sequencing could undisputedly offer a direct link between phenotype and genotype of cells, which is especially significant in microbiology.

It has been reported that mini‐metagenomics, which starts from tens to thousands of cells and employs the single cell technology could improve amplification and help the genome assembly from the sequencing data, leading to higher hit of target gene and coverage as well as the integrity of assembled genome. In combination with advanced contig binning methods, multiple genomes can be reconstructed from mini‐metagenomic data of microbiota (McLean *et al*., 2013; Yao *et al*., 2017; Yu *et al*., 2017). In this study, RACE isolated 30 cells for mini‐metagenomic sequencing process, which can be served as blueprints for the whole genome assembly using advanced binning methods.

Several Raman activated cell sorting methods have been established, including Raman tweezers, RACE (Song *et al*., 2017), microfluidic flow sorting (RACS, RADS) (Zhang *et al*., 2015b; McIlvenna *et al*., 2016; Wang *et al*., 2017). RACE with single cell genomics can provide direct link between phenotype and genotype of the cell. However, the technical problem is that genome coverage from single cells is low due to extremely small DNA template, severe nonspecific and biased amplification for single cells (Song *et al*., 2017). Various attempts have been made to improve single‐cell sequencing (Rinke *et al*., 2013; Stepanauskas *et al*., 2017); however high coverage of single‐cell genome continues to be difficult to achieve.

The RACE is based on laser induced forward transfer (LIFT) principle, in which, most of the laser energy was absorbed by the coating material to push forward the cells (Song *et al*., 2017). However, the LIFT process may affect bacterial viability and so far we are unable to culture cells after RACE sorting, although there are some reports that cells were still viable after the LIFT (Ringeisen *et al*., 2004; Hopp *et al*., 2005; Barron *et al*., 2005).

### Pelagibacterales (SAR11) is able to fix CO_2_ in the seawater

In this study, we applied RACE to obtain mini‐metagenomes from functional bacteria, which served as the reference for the reconstruction of nearly complete genomes of two CO_2_ fixing bacteria *Synechococcus* spp. and *Pelagibacter* spp. originating from the Yellow Sea in China. The gene and pathway analyses confirm that *Synechococcus* spp. and *Pelagibacter* spp. have the necessary genes for functions such as CO_2_ fixing, carotenoid synthesis and light harvesting, which was in very good agreement with Raman profiling.

Cyanobacteria, including *Prochlorococcus* and *Synechococcus*, are the most abundant photosynthetic organisms on Earth (Scanlan *et al*., 2009) and are able to fix CO_2_ using sunlight as energy source. *Synechococcus* spp. was found to be one of the main CO_2_ fixing bacteria in the seawater, and the reconstructed genome shows that it contained all the genes necessary for this ecological function.

It has been reported that Pelagibacterales play an important role in marine carbon and nutrient cycling (Sowell *et al*., 2009; Ottesen *et al*., 2014). Metagenomic DNA sequencing suggested that globally SAR11 bacteria (Pelagibacterales) are estimated to account for 25% of all plankton (Giovannoni *et al*., 2005; Giovannoni, 2017). Despite its global abundance, our understanding of ecological role of SAR11 is limited due to the difficulty of cultivation and lack of suitable tools. All SAR11 members are thought to be chemoheterotrophic, obtaining its energy from the oxidation of organic compounds (Giovannoni, 2017). PR is a light driven proton pump, which couples with ATPase to help cells generate ATP for survival (DeLong and Beja, 2010; Gomez‐Consarnau *et al*., 2010). In this study, Pelagibacterales was found to be the dominant bacteria (25.8%) in the low organic matter sea water (Supporting Information Fig. S7). Interestingly, *Pelagibacter* spp. was sorted by RACE, indicating it contained carotenoid and was labelled with ^13^C carbon. The ‘reconstructed’ draft genome shows that *Pelagibacter* spp. contains self‐sufficient PR synthesis genes for light harvesting and key genes for anaplerotic CO_2_ fixation.

It is intriguing whether these PR‐containing *Pelagibacter* spp. were able to fix CO_2_ or crossly fed ^13^C from other bacteria. It was observed that both *Synechococcus* spp. and *Pelagibacter* spp. show the same extent ^13^C‐Raman shift at the same time (Fig. 3 and Supporting Information Fig. S4), which indicates the same level of ^13^C content in cells (Li *et al*., 2012b). In this study, both *Synechococcus* spp. and *Pelagibacter* spp. were dominant bacteria (Fig. 2) in the pelagic zone with low organic matter (COD = 1.05 mg l^−1^; Supporting Information Table S1). To grow and make dominant with limited organics, *Pelagibacter* spp. could fix CO_2_, given that genetically it was genetically equipped with an intact light‐harvesting PR (but no chlorophyll) and CO_2_ fixing (anaplerotic reactions) machinery. It has been reported that anaplerotic CO_2_ fixation in PR containing *Dokdonia* sp. MED134 provided up to one‐third of the cell carbon in the light (Palovaara *et al*., 2014). Hence, it is highly likely that *Pelagibacter* spp. also contribute to CO_2_ fixation in open sea.

In summary, this study demonstrates that metagenomics aided RACE is able to establish a reliable link between the phenotype and the genotype of key functional bacteria in microbial community, addressing the fundamental questions and paving the way for comprehensive dissection of microbial community.

## Experimental procedures

### Seawater sampling and preparation

Two 10 L seawater samples were collected by Schindler samplers from the euphotic zone of the Yellow Sea, China (the geographical position is shown in Supporting Information Fig. S2). The seawater was transported using portable cooler with ice to laboratory for analysis. All samples were prefiltered through 8 μm pore size filter membranes using a peristaltic pump when being decanted from the sampler.

The original seawater sample was served as a primary control (without incubation). One liter of seawater was taken and spiked with ^12^C or ^13^C NaHCO_3_ with a final concentration of 2 mM, and the control was not treated with any chemicals. The samples were incubated in closed bottles at room temperature for 5 days with natural light. The four sets of samples, including primary control, incubation control, ^12^C or ^13^C NaHCO_3_ spiking treatments, were all in triplicate. After pretreatments, cells in those samples mentioned above were harvested by 0.22 μm pore size membrane filtration using a peristaltic pump and then the cells were sealed and stored at −80°C freezer until DNA extraction.

After incubation, cells in samples spiked with ^12^C and ^13^C NaHCO_3_ were enriched by passing 70 ml seawater through the Centricon® Plus‐70 Ultracel PL‐100 (Merck Millipore, Billerica, USA) respectively. Then, the cells were used for Raman activated cell sorting based on functions of ^13^C NaHCO_3_ utilization.

### ‘All‐in‐one’ device for Raman‐activated cell ejection (RACE)

RACE analysis in this study was performed using a custom‐made ‘all‐in‐one’ device, which represents a significant improvement over our previously reported system (Song *et al*., 2017). The device consists of a transparent ejection slide plus a sampling and cell collection chip (Supporting Information Fig. S1). The transparent ejection slide was fabricated by coating a thin layer of indium tin oxide on the surface of a 1 mm thick quartz substrate, which formed an optical transparent film of 100 nm thickness over the quartz slide, enabling the acquisition of Raman scattering signals and the ejection of cells of interest without the need of opening system for slide turnover (Supporting Information Fig. S1). This improvement would significantly reduce the risk of contamination. The sampling chip was composed of an elastomer polydimethylsiloxane (PDMS) layer with arrayed hollow holes (1 mm in diameter), which was attached onto the transparent ejection slide to form sample wells (Supporting Information Fig. S1). The cell collection chip was fabricated by irreversibly bonding another thicker PDMS layer with holes to a glass cover chip, forming wells (2 mm in diameter) for receiving the sorted cells and for holding reagents for subsequent cell lysis. The wells in cell collection chip were aligned with the wells in sampling chip, avoiding cross contamination.

### Single‐cell Raman micro‐spectroscopy

The cell samples of 2 μl were immediately loaded into the specified mini‐wells in the ‘all‐in‐one’ system (Supporting Information Fig. S1) and air‐dried prior to Raman analysis. Sample observation and Raman signal acquisition were achieved by using a modified Horiba LabRam HR system (Hesen Ltd, Shanghai, China), which was equipped with a confocal microscope with a 50× PL magnifying dry objective (NA = 0.55, BX41; Olympus UK Ltd., Southall, UK) and a 532 nm Nd:YAG laser (Ventus; Laser Quantum Ltd, Stockport, UK). The laser power out of the objective was approximately 5 mW and Raman signals collection was by a Newton EMCCD (Andor, Belfast, UK) utilizing a 1600 × 200 array of 16 μm pixels with thermoelectric cooling down to −70°C for negligible dark current. LabSpec5 (Horiba Scientific, France) software was used to control the Raman system and acquire spectra. A 600 mm^−1^ grating was set for the measurements, resulting in a spectral resolution of ∼ 1 cm^−1^ with 1600 data points. Acquisition of each spectrum was performed within one second.

### Isolation of ^13^C cells from the seawater sample by RACE

All Raman spectra were recorded and normalized with the LabSpec 5 software. The positions of SCRS bands from carotenoid‐containing cells were determined and analysed to establish a relationship between the red shift and ^13^C absorption phenomenon in cells. The Raman spectra of carotenoid‐containing cells from ^12^C labelling sample were used as controls. Cells with ^13^C shift in SCRS were isolated using a 532 nm pulsed laser (Alphalas GmbH, Goettingen, Germany), as described previously (Song *et al*., 2017). After cells were measured and ejected one‐by‐one into the collection chip, the whole device was moved into a laminar hood. The cell collection chip was then carefully detached from the device, and buffer (Qiagen, Germantown, MD, USA) was added into the well for cell lysis.

### Nucleotide sequence accession numbers

The sequence data reported in this study have been deposited to NCBI SRA database with bioproject accession PRJNA407379.

## Author contributions

WEH conceived the idea. WEH, JX, XJ and HG designed the research. LX provided seawater samples. XJ, HG and XS performed the Raman microspectroscopy, RACE, DNA amplification, library‐generation and sequencing. HG designed and manufactured the RACE device. YG, XJ and JX performed metagenome sequence analysis. WEH, XJ, HG, YG and JX analyzed the data. YJ, YS and IPT provided critical suggestions. WEH, JX, XJ, HG and YG wrote the article.

## Supporting information

Additional supporting information may be found in the online version of this article at the publisher's web‐site

**Table S1**.  Basic properties of the seawater sampled from Yellow Sea of China.
**Table S2**.  Numbers of 16S rRNA sequencing reads for microbial diversity analysis of each sample.
**Table S3**.  Primers used in 16S rRNA PCR amplification.
**Table S4**.  Sequencing and assembly statistics for the functional mini‐metagenomes and shotgun metagenomes.
**Table S5**.  The statistics of the predicted genes in mini‐metagenome sequencing in this study.
**Table S6**.  The KEGG pathways related to key metabolism pathways for gene annotation and their corresponding gene counts.
**Table S7**.  Protein similarities in three KEGG modules (beta‐carotene biosynthesis, reductive pentose phosphate cycle and reductive citrate cycle) between mini‐metagenomic data and shotgun metagenomic data for *Chroococcales* and *Pelagibacterales*.

**Fig. S1**.  Illustration of the ‘all‐in‐one’ device assembly.
A. Transparent ejection slide.
B. Sampling chip.
C. Cell collection chip.
**Fig. S2**.  Geographic location of sampling site in Yellow Sea.
The label ‘L 01’ represents the location position of sampling site.
**Fig. S3**.  Scheme of Raman identification and sorting of CO_2_‐fixing microbes with ‘all‐in‐one’ integrated device.
A. Acquisition of single cell Raman spectra and CO_2_‐fixing microbes identification.
B. Sorting cells of interest by laser ejection.
C. Shifts of the carotenoid Raman bands in single cell Raman spectra of *Synechococcus* spp. PCC7002, indicating ^13^C incorporation into the cell when cells were incubated with ^13^C NaHCO_3_.
**Fig. S4**.  Raman spectra of carotenoids containing cells in the seawater which were incubated in closed bottles at room temperature at different times.
A. The average Raman spectra of cells treated with ^13^C NaHCO_3_ and ^12^C NaHCO_3_, respectively, at time *t* = 0 days.
B. The average Raman spectra of cells treated with ^13^C NaHCO_3_ and ^12^C NaHCO_3_, respectively, at time *t* = 3 days.
C. The average Raman spectra of cells treated with ^13^C NaHCO_3_ and ^12^C NaHCO_3_, respectively, at time *t* = 5 days.
D. The average Raman spectra of cells treated with ^13^C NaHCO_3_ and ^12^C NaHCO_3_, respectively, at time *t* = 7 days.
E. The average Raman spectra of cells treated with ^13^C NaHCO_3_ and ^12^C NaHCO_3_, respectively, at time *t* = 10 days.
**Fig. S5**.  Alpha diversity comparisons across four different treatments of the seawater: red bars – ^12^C NaHCO_3_ amended sample (12C); blue bars – ^13^C NaHCO_3_ amended sample (13C); green bars – control sample without NaHCO_3_ (C_free); purple bars – original seawater sample control (primary).
A. Box plot showing the variation of observed OTUs.
B. Box plot showing the variation of Chao1 index.
C. Box plot showing the variation of Simpson index.
D. Box plot showing the variation of Shannon index.
**Fig. S6**.  Agarose gel images of the multiple displacement amplifications (MDAs) and 16S rRNA gene validation processes.
A. Agarose gel image of the DNA products after MDAs showing high molecular weight DNA.
B. Agarose gel image of the PCR products of 16S rRNA gene from isolated single marine cells.
Lane M, DNA ladder; Lane N, negative control for PCR; Lane N1, negative control for ddH_2_O and REPLI‐g sc Master Mix (contains REPLI‐g sc Reaction buffer and REPLI‐g sc DNA Polymerase); Lane N2, negative control for ddH_2_O, REPLI‐g sc Master Mix, Stop solution and lysis buffer (contains buffer DLB and DTT); Lanes 1–5, 7 and 8, 30 ejected target cells in each sample; Lane 6, thirty random ejected cells; Lane 9, no cells were ejected; Lane P, Lane P1, positive control for PCR.
**Fig. S7**.  Heat tree visualization of the structure of marine bacterial community based on 16S rDNA sequencing.
In the heat tree, size and color of nodes and edges are correlated with the abundance of organisms in the microbial community.
**Fig. S8**.  GC distributions of metagenomic reads which were mapped to the binned genomes of *Synechococcus* spp. (A) and *Pelagibacter* spp. (B) from shotgun metagenomes.
The two curves were smooth and normally distributed, which is consistent with the absence of significant contamination. The central peak in each curve (red) was close to the average GC content of the binned draft genomes and also to the sequenced reference genomes of identical taxonomic classification.
**Fig. S9**.  Reconstructed ‘carotenoid biosynthesis’ pathway by metagenomics‐aided RACE (A) and mini‐metagenome (B) in Yellow sea.
The known pathways were obtained from the KEGG database. Green text represents proteins found only in *Synechococcus* spp.; yellow text represents proteins found only in *Pelagibacter* spp.; and red text represents proteins found in both.
**Fig. S10**.  Reconstructed ‘carbon fixation in photosynthetic organisms’ pathway by metagenomics aided RACE (A) and mini‐metagenome (B) in Yellow sea.
The known pathways were obtained from the KEGG database. Green text represents proteins found only in *Synechococcus* spp.; yellow text represents proteins found only in *Pelagibacter* spp.; and red text represents proteins found in both.
**Fig. S11**.  Reconstructed ‘carbon fixation pathways in prokaryotes’ by metagenomics aided RACE (A) and mini‐metagenome (B) in Yellow sea.
The known pathways were obtained from the KEGG database. Green text represents proteins found only in *Synechococcus* spp.; yellow text represents proteins found only in *Pelagibacter* spp.; and red text represents proteins found in both.
**Fig. S12**.  Reconstructed ‘photosynthesis’ pathway by metagenomics aided RACE (A) and mini‐metagenome (B) in Yellow sea.
The known pathways were obtained from the KEGG database. Green text represents proteins found only in *Synechococcus* spp.; yellow text represents proteins found only in *Pelagibacter* spp.; and red text represents proteins found in both.
**Fig. S13**.  Reconstructed ‘photosynthesis‐antenna proteins’ pathway by metagenomics aided RACE (A) and mini‐metagenome (B) in Yellow sea.
The known pathways were obtained from the KEGG database. Green text represents proteins found only in *Synechococcus* spp.; yellow text represents proteins found only in *Pelagibacter* spp.; and red text represents proteins found in both.
**Fig. S14**.  Reconstructed ‘porphyrin and chlorophyll metabolism’ pathway by metagenomics aided RACE (A) and mini‐metagenome (B) in Yellow sea.
The known pathways were obtained from the KEGG database. Green text represents proteins found only in *Synechococcus* spp.; yellow text represents proteins found only in *Pelagibacter* spp.; and red text represents proteins found in both.
**Fig. S15**.  Reconstructed ‘terpenoid backbone biosynthesis’ pathway by metagenomics aided RACE (A) and mini‐metagenome (B) in Yellow sea. The known pathways were obtained from the KEGG database. Green text represents proteins found only in *Synechococcus* spp.; yellow text represents proteins found only in *Pelagibacter* spp.; and red text represents proteins found in both.

**File S1**.  List of carbon metabolism‐related genes and their annotations in the mini‐metagenomic and the shotgun metagenomic data.

**File S2**.  Identified functional genes in metabolic pathways from the mini‐metagenome data and their corresponding genes in the shotgun metagenomic data.

**File S3**.  Identified proteorhodopsin (PR) genes from metagenomic bins of *Pelagibacter* spp.
