# Supplementary material for: Raman‐activated cell sorting and metagenomic sequencing revealing carbon‐fixing bacteria in the ocean
Source: Environ Microbiol. 2018 Jul 2;20(6):2241–55. doi: 10.1111/1462-2920.14268 (PMC6849569; doi:10.1111/1462-2920.14268)
Supplement: Supplementary file 1 — Table S1. Basic properties of the seawater sampled from Yellow Sea of China. Table S2. Numbers of 16S rRNA sequencing reads for microbial diversity analysis of each sample. Table S3. Primers used in 16S rRNA PCR amplification. Table S4. Sequencing and assembly statistics for the functional mini‐metagenomes and shotgun metagenomes. Table S5. The statistics of the predicted genes in mini‐metagenome sequencing in this study. Table S6. The KEGG pathways related to key metabolism pathways for gene annotation and their corresponding gene counts. Table S7. Protein similarities in three KEGG modules (beta‐carotene biosynthesis, reductive pentose phosphate cycle and reductive citrate cycle) between mini‐metagenomic data and shotgun metagenomic data for Chroococcales and Pelagibacterales. [file EMI-20-2241-s001.docx]

**Supplementary information**

**Raman-activated cell sorting and metagenome sequencing reveal carbon-fixing bacteria in the ocean**

Xiaoyan Jing^1,2,3,†^, Honglei Gou^1,3†^, Yanhai Gong^1,3†^, Xiaolu Su^1,3^, La Xu^4^, Yuetong Ji^1,3^, Yizhi Song^2^, Ian P. Thompson^2^, Jian Xu^1,3,^*, Wei E. Huang^2,^*

^1^Single-Cell Center, CAS Key Laboratory of Biofuels and Shandong Key Laboratory of Energy Genetics, Qingdao Institute of BioEnergy and Bioprocess Technology, Chinese Academy of Sciences, Qingdao, Shandong, China.

^2^Department of Engineering Science, University of Oxford, Parks Road, OX1 3PJ, Oxford, United Kingdom.

^3^University of Chinese Academy of Sciences, Beijing, China.

^4^Disease and Fishery Drugs Research Center, Marine Biology Institute of Shandong Province, Qingdao, Shandong, China.

^†^These authors contributed equally to this work.

*Corresponding author:

Wei E. Huang and Jian Xu

Email: [wei.huang@eng.ox.ac.uk](mailto:wei.huang@eng.ox.ac.uk) and [xujian@qibebt.ac.cn](mailto:xujian@qibebt.ac.cn)

**Materials and methods**

***Measurement of seawater environmental parameters***

Seawater and relevant environmental parameters (**Table S1**), including water temperature (T), salinity, pH, dissolved oxygen (DO) and chemical oxygen demand (COD) were recorded *in situ* employing portable devices (YSI Pro_Plus, Yellow Springs, OH, USA). Other environmental parameters, such as total phosphorus (TP), total nitrogen (TN), [orthophosphate](javascript:void(0);) (PO_4_^3-^-P) and [chlorophyll](javascript:void(0);) *a* (Chl-*a*) were assayed in laboratory according to Standard Methods (APHA, 1998). All data were collected in accordance with the approved Chinese regulations and standards.

***^13^C labelling of Synechococcus* sp. PCC 7002**

*Synechococcus* sp. PCC 7002 was used as reference of Raman shift for ^13^CO_2_-fixing bacteria. *Synechococcus* sp. PCC 7002 was grown in modified medium A (50 mg l^-1^ KH_2_PO_4_, 18 g l^-1^ NaCl, 5.0 g l^-1^ MgSO_4_•7H_2_O, 0.37 g l^-1^ CaCl_2_•2H_2_O, 0.60 g l^-1^ KCl, 32 mg l^-1^ Na_2_EDTA•2H_2_O, 8.0 mg l^-1^ FeCl_3_•6H_2_O, 34 mg l^-1^ H_3_BO_3_, 4.3 mg l^-1^ MnCl_2_•4H_2_O, 0.32 mg l^-1^ ZnCl_2_, 30 μg l^-1^ MoO_3_, 3.0 μg l^-1^ CuSO_4_•5H_2_O, 12 μg l^-1^ CoCl_2_•6H_2_O, 4.0 μg l^-1^ cobalamin, and 8.3 mM Tris aminomethane) supplemented with 1 mg NaNO_3_ ml^-1^. ^13^C-labelled sodium bicarbonate (Sigma Aldrich, St.Louis, ML, USA) were spiked into the medium with a final concentration of 5 mM as the sole carbon source. The cells were incubated for 5 days in a shaking light incubator set at 150 rpm and 38 ^o^C temperature, and light intensity of 100 μmol m^-2^ sec^-1^. The cells were then harvested for Raman measurement.

***Multiple displacement amplification***

The REPLI-g Single Cell Kit (Qiagen, Germantown, MD, USA) was used for the single cell amplification according to the manufacture's instruction. Cell lysis was carried out at 65℃ for 15 min with 2 µl lysis buffer for each sample on-chip (**Fig. S1**), followed by addition of 1 µl stop solution to neutralize the lysis buffer. Then the DNA from each well was transferred from the receiving well (**Fig. S1**) to PCR tubes, respectively. REPLI-g sc Reaction Buffer and REPLI-g sc DNA Polymerase were added and the mixture was incubated at 30℃ for 8 hours with 70℃ hot-lid temperature for MDA reactions. Blank control (without any cells) was also included to detect and quantify potential contamination. After that, the MDA products were processed for 16S rRNA gene PCR analysis and then high throughput sequencing.

***Library construction and next generation sequencing***

***For functional mini-metagenomic sequencing:*** The target MDA products were treated with S1 Nuclease (Thermo Fisher Scientific, Waltham, MA, USA) to degrade the single-stranded nucleic acids, and then purified by Agencourt AMPure XP Beads (Beckman Coulter, Brea, CA, USA). Samples were quantified using a Qubit® 2.0 Fluorometer (Invitrogen, Carlsbad, CA, USA). For each sample, 132 ng of the purified MDA product was sheared randomly by a Covaris® M220 Focused-ultrasonicator™ (Thermo Fisher Scientific, Waltham, MA, USA), and the fragments of approximately 270 bp were selected. Next-generation sequencing libraries were constructed by Accel-NGS® 2S Plus DNA Library kit (Swift Biosciences, Ann Arbor, MI, USA) according to the manufacturer's protocol. They were then validated using an Agilent 2100 Bioanalyzer (Agilent Technologies, Palo Alto, CA, USA) and quantified by a Qubit® 2.0 Fluorometer. Finally, 2 × 150 bp paired-end reads were sequenced on the Illumina HiSeq 2500 platform (GeneWiz Biotechnology Co., Ltd).

***For shotgun metagenomic sequencing:*** The DNA sample for metagenomic sequencing was extracted by TIANamp Soil DNA Kit (Tiangen, China, referred to as TG), and then quantified using a Qubit® 2.0 Fluorometer (Invitrogen, Carlsbad, CA, USA). The amount of 250 ng genomic DNA was randomly fragmented to < 500 bp by sonication (Covaris S220) for library construction. Next generation sequencing library preparations were constructed following the manufacturer’s protocol (NEBNext® Ultra™ DNA Library Prep Kit for Illumina®). Then these libraries were cleaned up using AxyPrep Mag PCR Cleanup Kit (Axygen, Corning, Tewksburym, MA, USA), validated using an Agilent 2100 Bioanalyzer (Agilent Technologies, Palo Alto, CA, USA), and quantified by a Qubit2.0 Fluorometer (Invitrogen, Carlsbad, CA, USA). Finally, 2 x 150 paired-end (PE) sequencing runs were performed on each library pool on the Illumina HiSeq 2500 platform.

***For 16S rRNA sequencing:*** The filter membranes were aseptically cut into quarters for DNA extraction using TIANamp Soil DNA Kit (Tiangen, China). DNA was quantified using the Qubit® 2.0 Fluorometer (Invitrogen, Carlsbad, CA, USA). For each sample, 30-50 ng DNA was used to generate amplicons using a MetaVx™ Library Preparation kit (Genewiz, South Plainfield, NJ, USA). V3 and V4 hypervariable regions of prokaryotic 16S rDNA were selected for generating amplicons and subsequent taxonomy analysis. The v3 and v4 regions were amplified using forward primers with the sequence “CCTACGGRRBGCASCAGKVRVGAAT” and reverse primers with the sequence “GGACTACNVGGGTWTCTAATCC”. First-round PCR products were used as templates for the second-round amplicon enrichment PCR. At the same time, indexed adapters were added to the ends of the 16S rDNA amplicons to generate indexed libraries ready for downstream NGS sequencing on Illumina MiSeq. DNA libraries were validated by Agilent 2100 Bioanalyzer (Agilent Technologies, Palo Alto, CA, USA), and quantified by Qubit® 2.0 Fluorometer. Then, DNA libraries were multiplexed and loaded on an Illumina MiSeq instrument according to the manufacturer’s instructions (Illumina, San Diego, CA, USA). Sequencing was performed using a 2 x 300 paired-end (PE) configuration.

***Data analysis***

***For functional mini-metagenomic sequencing******:*** *Quality control and contig assembly.* Raw sequencing reads were firstly quality-checked using FastQC (version 0.11.3), then three steps were performed to obtain a high-quality clean dataset using Trimmomatic (version 0.36, PE mode with parameters “ILLUMINACLIP:adapters.fa:3:30:10:3:TRUE SLIDINGWINDOW :10:25 MAXINFO:120:0.3 LEADING:30 TRAILING:30 MINLEN:30”) (Bolger et al., 2014) : (1) removing primers and adapter contamination; (2) accepting reads only with a mean quality score above 25 using a sliding window of 10 bp; (3) keeping the minimum read length of 30 bp. Then, assemblies were produced by SPAdes (version 3.10.1; (Bankevich et al., 2012)) with the *--sc* and *--careful* flags asserted.

*Contigs binning and genome quality assessment.* Blobtools (version 0.9.19.4) (Kumar et al., 2013) was used for the visualization of mini-metagenome assemblies through TAGC (Taxon-annotated GC-Coverage) plots. To increase the hit rate of environmental metagenomes, e-value of 1e-6 was used for taxonomic annotation. Putative contaminated contigs were removed based on the homology annotation via comparison with those generated from negative controls. Besides, ACDC (Lux et al., 2016), which uses sophisticated dimensionality reduction (Barnes-Hut t-SNE) and clustering methods (CC and DIP) to the tetramer profiles to test contamination in single-cell genome data and to differentiate between different species in a given sample, was also used in our study. The quality of genome bins was quantified *via* CheckM (version 1.0.7; (Parks et al., 2015)), which is an automated method for assessing the completeness and degree of contamination using a broader set of marker genes within a reference genome tree.

*Gene prediction and functional annotation.* Prokka (version 1.11, with minor modification to allow partial gene prediction, and with "--metagenome" parameter for metagenomes and highly fragmented genomes) was used to predict and annotate genes in the resulting contigs which were larger than 200 bp. Functional orthologs (KO terms) of protein-coding genes were predicted using online service GhostKOALA (Kanehisa et al., 2016), and the associated Pathways and Enzymes were extracted using custom scripts with the aid of KEGG API. The specific pathways reconstructed were visualized using the “Search & Color Pathway” tool from KEGG Mapper. To find proteorhodopsin (PR) genes, BLASTp was used with e-value cutoff of 10^-6^ from a custom database built by combining Swiss-Prot database and full-length PR genes from TrEMBL database.

***For shotgun metagenomic sequencing:*** *Quality control and contig assembly.* The same procedures as those for functional mini-metagenomic sequencing were performed for shotgun metagenomic sequencing, while MEGAHIT (version 1.1.1) was used to produce assembly on a NVIDIA K40c GPU board.

*Contigs binning and genome quality assessment.* Blobtools (version 0.9.19.4; (Kumar et al., 2013)) was used as well for the visualization of the metagenome assembly through TAGC (Taxon-annotated GC-Coverage) plots and to bin contigs according to homology annotations at different taxonomic levels. Besides, MetaBAT (version 0.32.4) was used to bin those contigs with 30 times of bootstrapping and minimum contig lengths of 1500 bp. The quality of genome bins was quantified *via* CheckM (version 1.0.7; (Parks et al., 2015) as above.

*Gene prediction and functional annotation for the target.* Similar approaches as in mini-metagenome were employed to predict and annotate genes in assembled contigs from the metagenomic data for the target.

*Link mini-metagenomes with shotgun metagenomes.* Contigs of mini-metagenomes which belong to order *Chroococcales* and *Pelagibacterales* were examined to the genus level. For *Chroococcales*, most of the contigs were *Synechococcus*; while for *Pelagibacterales*, *Candidatus Pelagibacter* and *Pelagibacteraceae-undef* were dominate. Then, contigs with corresponding genus level annotations were extracted from shotgun metagenomes.

***For the 16S rRNA sequencing:*** The software package of QIIME (version 1.9.1) (Caporaso et al., 2010) was used for 16S rRNA data analysis. The forward and reverse reads were joined and assigned to samples based on barcode and truncated by cutting off the barcode and primer sequence. Quality filtering on joined sequences was performed and sequences which did not fulfill the following criteria were discarded: sequence length > 200bp, no ambiguous bases and mean quality score >= 20. Then the chimeric sequences were removed using UCHIME algorithm (Edgar et al., 2011). Putative contaminants (comparing candidate sequences against the Greengenes database, the August 2013 release) were removed from datasets, as were singletons. Subsequently, the remaining high-quality reads were grouped into operational taxonomic units (OTUs) using the uclust algorithm (Edgar, 2010), and aligned using default parameters against the Greengenes database (DeSantis et al., 2006). Representative sequences for the shared OTUs, as defined by 97% similarity, were obtained. Alpha diversity indexes, including Good's coverage (sample coverage; (Bik et al., 2010)), Chao1 (an OTU richness index; (Hughes et al., 2001)), Shannon index (a diversity index that takes into account abundance and evenness; (Spellerberg and Fedor, 2003)) and Simpson index (a diversity index that describes the probability that a second individual drawn from a population should be of the same species as the first; (Chao and Lee, 1992; Morris et al., 2014)), were respectively calculated and then analyzed via the Kruskal-Wallis test. Community taxonomic diversity data were visualized using the R package of Metacoder (version 0.1.3; (Foster et al., 2017)). Relative abundances of the bacterial taxa at the phylum, class, order, family, genus and species levels were calculated and compared, respectively.

**References**

APHA (1998) Standard Method for the Examination of Water and Wastewater. *20th Edition, American Public Health Association, Washington DC,*.

Bankevich, A., Nurk, S., Antipov, D., Gurevich, A.A., Dvorkin, M., Kulikov, A.S. et al. (2012) SPAdes: a new genome assembly algorithm and its applications to single-cell sequencing. *J Comput Biol* **19**: 455-477.

Bik, E.M., Long, C.D., Armitage, G.C., Loomer, P., Emerson, J., Mongodin, E.F. et al. (2010) Bacterial diversity in the oral cavity of ten healthy individuals. *ISME J* **4**: 962-974.

Bolger, A.M., Lohse, M., and Usadel, B. (2014) Trimmomatic: a flexible trimmer for Illumina sequence data. *Bioinformatics* **30**: 2114-2120.

Caporaso, J.G., Kuczynski, J., Stombaugh, J., Bittinger, K., Bushman, F.D., Costello, E.K. et al. (2010) QIIME allows analysis of high-throughput community sequencing data. *Nat Methods* **7**: 335-336.

Chao, A., and Lee, S.-M. (1992) Estimating the Number of Classes via Sample Coverage. *J Am Stat Assoc* **87**: 210-217.

DeSantis, T.Z., Hugenholtz, P., Larsen, N., Rojas, M., Brodie, E.L., Keller, K. et al. (2006) Greengenes, a Chimera-Checked 16S rRNA Gene Database and Workbench Compatible with ARB. *Appl Environ Microbiol* **72**: 5069-5072.

Edgar, R.C. (2010) Search and clustering orders of magnitude faster than BLAST. *Bioinformatics* **26**: 2460-2461.

Edgar, R.C., Haas, B.J., Clemente, J.C., Quince, C., and Knight, R. (2011) UCHIME improves sensitivity and speed of chimera detection. *Bioinformatics* **27**: 2194-2200.

Foster, Z.S.L., Sharpton, T.J., and Grünwald, N.J. (2017) Metacoder: An R package for visualization and manipulation of community taxonomic diversity data. *PLOS Comput Biol* **13**: e1005404.

Hughes, J.B., Hellmann, J.J., Ricketts, T.H., and Bohannan, B.J.M. (2001) Counting the Uncountable: Statistical Approaches to Estimating Microbial Diversity. *Appl Environ Microbiol* **67**: 4399-4406.

Kanehisa, M., Sato, Y., and Morishima, K. (2016) BlastKOALA and GhostKOALA: KEGG Tools for Functional Characterization of Genome and Metagenome Sequences. *J Mol Biol* **428**: 726-731.

Kumar, S., Jones, M., Koutsovoulos, G., Clarke, M., and Blaxter, M. (2013) Blobology: exploring raw genome data for contaminants, symbionts and parasites using taxon-annotated GC-coverage plots. *Front Genet* **4**: 237.

Lux, M., Krüger, J., Rinke, C., Maus, I., Schlüter, A., Woyke, T. et al. (2016) acdc – Automated Contamination Detection and Confidence estimation for single-cell genome data. *BMC Bioinformatics* **17**: 543.

Morris, E.K., Caruso, T., Buscot, F., Fischer, M., Hancock, C., Maier, T.S. et al. (2014) Choosing and using diversity indices: insights for ecological applications from the German Biodiversity Exploratories. *Ecol and Evol* **4**: 3514-3524.

Parks, D.H., Imelfort, M., Skennerton, C.T., Hugenholtz, P., and Tyson, G.W. (2015) CheckM: assessing the quality of microbial genomes recovered from isolates, single cells, and metagenomes. *Genome Res* **25**: 1043-1055.

Spellerberg, I.F., and Fedor, P.J. (2003) A tribute to Claude Shannon (1916–2001) and a plea for more rigorous use of species richness, species diversity and the ‘Shannon–Wiener’ Index. *Glob Ecol and Biogeogr***12**: 177-179.

**Supplemental Tables and Figures**

**Supplementary Table S1. Basic properties of the seawater sampled from Yellow Sea of China.**

**Supplementary Table S2. Numbers of 16S rRNA sequencing reads for microbial diversity analysis of each sample.**

**Supplementary Table S3. Primers used in 16S rRNA PCR amplification.**

**Supplementary Table S4. Sequencing and assembly statistics for the functional mini-metagenomes and shotgun metagenomes.**

**Supplementary Table S5. The statistics of the predicted genes in mini-metagenome sequencing in this study.**

**Supplementary Table S6. The KEGG pathways related to key metabolism pathways for gene annotation and their corresponding gene counts.**

**Supplementary Table S7. Protein similarities in three KEGG modules (Beta-carotene biosynthesis, Reductive pentose phosphate cycle and Reductive citrate cycle) between mini-metagenomic data and shotgun metagenomic data for *Chroococcales* and *Pelagibacterales*.**

**Supplementary Figure S1.** **Illustration of the "All-In-One" device assembly. (A)** Transparent ejection slide; **(B)** Sampling chip; **(C)** Cell collection chip.

**Supplementary Figure S2.** **Geographic location of sampling site in Yellow Sea.** The label "L 01" represents the location position of sampling site.

**Supplementary Figure S3.** **Scheme of Raman identification and sorting of CO_2_-fixing microbes with "All-In-One" integrated device. (A)** Acquisition of single cell Raman spectra and CO_2_-fixing microbes identification; **(B)** Sorting cells of interest by laser ejection; **(C)** Shifts of the carotenoid Raman bands in single cell Raman spectra of *Synechococcus* spp. PCC7002, indicating ^13^C incorporation into the cell when cells were incubated with ^13^C-NaHCO_3_.

**Supplementary Figure S4.** **Raman spectra of carotenoids containing cells in the seawater which were incubated in closed bottles at room temperature at different times.** **(A)** the average Raman spectra of cells treated with ^13^C-NaHCO_3_ and ^12^C-NaHCO_3_ respectively, at time t=0 day; **(B)** the average Raman spectra of cells treated with ^13^C-NaHCO_3_ and ^12^C-NaHCO_3_ respectively, at time t=3 days; **(C)** the average Raman spectra of cells treated with ^13^C-NaHCO_3_ and ^12^C-NaHCO_3_ respectively, at time t=5 days; **(D)** the average Raman spectra of cells treated with ^13^C-NaHCO_3_ and ^12^C-NaHCO_3_ respectively, at time t=7 days; **(E)** the average Raman spectra of cells treated with ^13^C-NaHCO_3_ and ^12^C-NaHCO_3_ respectively, at time t=10 days.

**Supplementary Figure S5.** **Alpha diversity comparisons across four different treatments of the seawater:** red bars—^12^C-NaHCO_3_ amended sample (12C); blue bars—^13^C-NaHCO_3_ amended sample (13C); green bars— control sample without NaHCO_3_ (C_free); purple bars—Original seawater sample control (Primary). **(A)** Box plot showing the variation of Observed OTUs; **(B)** Box plot showing the variation of Chao1 index; **(C)** Box plot showing the variation of Simpson index; **(D)** Box plot showing the variation of Shannon index.

**Supplementary Figure S6.** **Agarose gel images of the multiple displacement amplifications (MDAs) and 16S rRNA gene validation processes. (A)** Agarose gel image of (MDAs) showing high molecular weight DNA. **(B)** Agarose gel image of the PCR products of 16S rRNA gene from isolated single marine cells. Lane M, DNA ladder; Lane N, negative control for PCR; Lane N1, negative control for ddH_2_O and REPLI-g sc Master Mix (contains REPLI-g sc Reaction buffer and REPLI-g sc DNA Polymerase); Lane N2, negative control for ddH_2_O, REPLI-g sc Master Mix, Stop solution and lysis buffer (contains buffer DLB and DTT); Lane 1-5,7 and 8, thirty ejected target cells in each sample; Lane 6, thirty random ejected cells; Lane 9, no cells were ejected; Lane P, Lane P1, positive control for PCR.

**Supplementary Figure S7. Heat tree visualization of the structure of marine bacterial community based on 16S rDNA sequencing.** In the heat tree, size and color of nodes and edges are correlated with the abundance of organisms in the microbial community.

**Supplementary Figure S8.** **GC distributions of metagenomic reads which were mapped to the binned genomes of *Synechococcus* spp. (A) and *Pelagibacter* spp. (B) from shotgun metagenomes.** The two curves were smooth and normally distributed, which is consistent with the absence of significant contamination. The central peak in each curve (red) was close to the average GC content of the binned draft genomes and also to the sequenced reference genomes of identical taxonomic classification.

**Supplementary Figure S9.** **Reconstructed 'carotenoid biosynthesis' pathway by metagenomics-aided RACE (A) and mini-metagenome (B) in Yellow sea.** The known pathways were obtained from the KEGG database. Green text represents proteins found only in *Synechococcus* spp.; Yellow text represents proteins found only in *Pelagibacter* spp.; Red text represents proteins found in both.

**Supplementary Figure S10.** **Reconstructed 'carbon fixation in photosynthetic organisms' pathway by metagenomics aided RACE (A) and mini-metagenome (B) in Yellow sea.** The known pathways were obtained from the KEGG database. Green text represents proteins found only in *Synechococcus* spp.; Yellow text represents proteins found only in *Pelagibacter* spp.; Red text represents proteins found in both.

**Supplementary Figure S11.** **Reconstructed 'carbon fixation pathways in prokaryotes' by metagenomics aided RACE (A) and mini-metagenome (B) in Yellow sea.** The known pathways were obtained from the KEGG database. Green text represents proteins found only in *Synechococcus* spp.; Yellow text represents proteins found only in *Pelagibacter* spp.; Red text represents proteins found in both.

**Supplementary Figure S12.** **Reconstructed 'photosynthesis' pathway by metagenomics aided RACE (A) and mini-metagenome (B) in Yellow sea.** The known pathways were obtained from the KEGG database. Green text represents proteins found only in *Synechococcus* spp.; Yellow text represents proteins found only in *Pelagibacter* spp.; Red text represents proteins found in both.

**Supplementary Figure S13.** **Reconstructed 'photosynthesis-antenna proteins' pathway by metagenomics aided RACE (A) and mini-metagenome (B) in Yellow sea.** The known pathways were obtained from the KEGG database. Green text represents proteins found only in *Synechococcus* spp.; Yellow text represents proteins found only in *Pelagibacter* spp.; Red text represents proteins found in both.

**Supplementary Figure S14.** **Reconstructed 'porphyrin and chlorophyll metabolism' pathway by metagenomics aided RACE (A) and mini-metagenome (B) in Yellow sea.** The known pathways were obtained from the KEGG database. Green text represents proteins found only in *Synechococcus* spp.; Yellow text represents proteins found only in *Pelagibacter* spp.; Red text represents proteins found in both.

**Supplementary Figure S15.** **Reconstructed 'terpenoid backbone biosynthesis' pathway by metagenomics aided RACE (A) and mini-metagenome (B) in Yellow sea.** The known pathways were obtained from the KEGG database. Green text represents proteins found only in *Synechococcus* spp.; Yellow text represents proteins found only in *Pelagibacter* spp.; Red text represents proteins found in both.

**Supplementary file 1.** **List of carbon metabolism-related genes and their annotations in the mini-metagenomic and the shotgun metagenomic data.**

**Supplementary file 2. Identified functional genes in metabolic pathways from the mini-metagenome data and their corresponding genes in the shotgun metagenomic data.**

**Supplementary file 3. Identified proteorhodopsin (PR) genes from metagenomic bins of *Pelagibacter* spp..**

**Supplementary Table S1. Basic properties of the seawater sampled from Yellow Sea of China.**

|  | Sample: #Primary* |
| --- | --- |
| Sample area | Laoshan Bay |
| Sample type | Seawater from euphotic zone |
| Location | Latitude (N), 36°24′; Longitude (E), 120°43′ |
| Collection time | October 14, 2016, 11:00am |
| Depth (m) | 3.300 |
| Temperature (℃) | 14.800 |
| Salinity (PSS-78) | 28.742 |
| Dissolved oxygen (mg/L) | 10.370 |
| Chemical oxygen demand (mg/L) | 1.050 |
| Total nitrogen (mg/L) | 0.660 |
| Total phosphorus (mg/L) | 0.039 |
| Orthophosphate (µg/L) | 6.560 |
| [Chlorophyll](javascript:void(0);) *a* (µg/L) | 0.388 |
| pH | 8.300 |

*primary sample control.

**Supplementary Table S2. Numbers of 16S rRNA sequencing reads for microbial diversity analysis of each sample.**

| Sample | Raw reads | Processed reads | Goods_  coverage(%) | Observed_OTUs | Shannon | Simpson | Chao1 |
| --- | --- | --- | --- | --- | --- | --- | --- |
| 12C_01 | 122,296 | 105,736 | 97.705 | 3029 | 5.794 | 0.979 | 3938 |
| 12C_02 | 121,513 | 104,978 | 97.691 | 3036 | 5.839 | 0.982 | 3984 |
| 12C_03 | 124,675 | 106,780 | 97.578 | 3109 | 5.830 | 0.980 | 4093 |
| 13C_01 | 122,462 | 105,442 | 97.590 | 3021 | 5.759 | 0.979 | 4062 |
| 13C_02 | 109,681 | 94,672 | 97.734 | 3006 | 5.857 | 0.982 | 3961 |
| 13C_03 | 116,342 | 99,763 | 97.746 | 2981 | 5.838 | 0.984 | 3847 |
| C_free_01 | 127,080 | 109,820 | 97.867 | 2976 | 5.809 | 0.980 | 3765 |
| C_free_02 | 125,620 | 107,909 | 97.740 | 3003 | 5.841 | 0.982 | 3929 |
| C_free_03 | 149,473 | 128,126 | 97.651 | 3114 | 5.940 | 0.985 | 4063 |
| Primary_01 | 72,306 | 57,332 | 97.869 | 2906 | 5.820 | 0.982 | 3645 |
| Primary_02 | 66,388 | 45,858 | 97.951 | 2849 | 5.721 | 0.979 | 3637 |
| Primary_03 | 108,880 | 90,369 | 97.874 | 2892 | 5.795 | 0.982 | 3708 |

**Supplementary Table S3. Primers used in 16S rRNA PCR amplification.**

| Description | Name | Primer Sequence (5'→ 3') |
| --- | --- | --- |
| 16S rRNA, partial | 519F | CAGCAGCCGCGGTRATA |
|  | 785R | GGACTACCVGGGTATCTAAKCC |
| 16S rRNA, V3-V4 region | F | CCTACGGRRBGCASCAGKVRVGAAT |
|  | R | GGACTACNVGGGTWTCTAATCC |

**Supplementary Table S4. Sequencing and assembly statistics for the functional mini-metagenomes and shotgun metagenomes.**

| Type | Mini-metagenome | | | | | | | | Shotgun metagenome |
| --- | --- | --- | --- | --- | --- | --- | --- | --- | --- |
| Type | **Total** | | **>= 200 bp** | | **>= 1,000 bp** | | **>= 1,500 bp** | | **>= 200 bp** |
| sample | **TET3** | **TET4** | **TET3** | **TET4** | **TET3** | **TET4** | **TET3** | **TET4** | **-** |
| Total sequences | 14,557 | 15,737 | 12,615 | 12,412 | 406 | 258 | 243 | 146 | 1,990,773 |
| Total bases | 4.72M | 4.49M | 4.53M | 4.15M | 0.93M | 0.59M | 734,944 | 460,796 | 1.67G |
| Min/Max lengths | 56/  8,327 | 56/  12,601 | 200/  8,327 | 201/  12,601 | 1,001/  8,327 | 1,000/  12,601 | 1,500/  8,327 | 1,503/  12,601 | 200/  501,864 |
| N50 | 292 | 273 | 307 | 284 | 2,767 | 2,828 | 3,226 | 3,613 | 1,028 |
| GC% | 43.49% | 42.74% | 43.82% | 43.54% | 44.15% | 38.07% | 43.96% | 37.02% | 46.48% |

**Supplementary Table S5. The statistics of the predicted genes in mini-metagenome sequencing in this study.**

| Sample | TET3 | TET4 |
| --- | --- | --- |
| Contig number | 3,620 | 2,658 |
| Bases | 1,804,857 | 1,217,685 |
| Gene | 4,098 | 3,102 |
| tmRNA | 1 | 2 |
| tRNA | 29 | 10 |
| CDS | 4,065 | 3,085 |
| rRNA | 3 | 5 |

**Supplementary Table S6. The KEGG pathways related to key metabolism pathways for gene annotation and their corresponding gene counts.**

| Functional Description | KEGG PATHWAY | KO Number | | | Gene Number | | |
| --- | --- | --- | --- | --- | --- | --- | --- |
|  |  | **TET3** | **TET4** | **Meta** | **TET3** | **TET4** | **Meta** |
| Carbon fixation | Carbon metabolism | 56 | 44 | 124 | 96 | 87 | 4831 |
|  | Carbon fixation in photosynthetic organisms | 10 | 10 | 23 | 14 | 20 | 961 |
|  | Carbon fixation pathways in prokaryotes | 16 | 17 | 36 | 26 | 31 | 1622 |
| Photosynthesis | Photosynthesis | 11 | 7 | 48 | 24 | 17 | 1332 |
|  | Photosynthesis-antenna proteins | 1 | 0 | 21 | 1 | 0 | 306 |
| Chlorophyll biosynthesis | Porphyrin and chlorophyll metabolism | 10 | 6 | 54 | 19 | 10 | 1466 |
| Carotenoid biosynthesis | Carotenoid biosynthesis | 6 | 0 | 10 | 10 | 0 | 209 |
|  | Terpenoid backbone biosynthesis | 5 | 7 | 20 | 8 | 8 | 584 |

**Supplementary Table S7. Protein similarities in three KEGG modules (β-carotene biosynthesis, Reductive pentose phosphate cycle and Reductive citrate cycle) between mini-metagenomic data and shotgun metagenomic data for *Chroococcales* and *Pelagibacterales*.**

| **Organism** | **Query (genes from mini-metagenome)** | **Query protein length** | **Annotation** | **Target (genes from shotgun metagenome)** | **Percent identity** | **Alignment length** | **E-value** | **Module** |
| --- | --- | --- | --- | --- | --- | --- | --- | --- |
| Chroococcales | Tf_00524 | 313 | All-trans-phytoene synthase | marine_60204 | 100 | 313 | 0 | β-carotene biosynthesis |
|  | Tf_00958 | 434 | Phytoene desaturase (neurosporene-forming) | marine_59955 | 99.08 | 434 | 0 |  |
|  | Tf_00958 | 434 | Phytoene desaturase (neurosporene-forming) | marine_115348 | 98.39 | 434 | 0 |  |
|  | Tf_01078 | 258 | 15-cis-phytoene desaturase | marine_60208 | 99.61 | 258 | 0 |  |
|  | Tf_01078 | 258 | 15-cis-phytoene desaturase | marine_114429 | 97.4 | 77 | 2.00E-50 |  |
|  | Tf_01825 | 111 | 15-cis-phytoene desaturase | marine_102935 | 96.4 | 111 | 5.00E-79 |  |
|  | Tf_01825 | 111 | 15-cis-phytoene desaturase | marine_81652 | 98.2 | 111 | 6.00E-79 |  |
|  | Tf_01825 | 111 | 15-cis-phytoene desaturase | marine_69828 | 95.5 | 111 | 2.00E-78 |  |
|  | Tf_01825 | 111 | 15-cis-phytoene desaturase | marine_107837 | 95.79 | 95 | 4.00E-66 |  |
|  | Tf_01825 | 111 | 15-cis-phytoene desaturase | marine_108205 | 96.77 | 93 | 3.00E-65 |  |
|  | Tf_01825 | 111 | 15-cis-phytoene desaturase | marine_73966 | 97.62 | 42 | 1.00E-23 |  |
|  | Tf_00095 | 334 | D-fructose 1,6-bisphosphatase class 2/sedoheptulose 1,7-bisphosphatase | marine_112843 | 99.4 | 334 | 0 | Reductive pentose phosphate cycle |
|  | Tf_00095 | 334 |  | marine_86922 | 98.5 | 334 | 0 |  |
|  | Tf_00095 | 334 |  | marine_81315 | 98.2 | 334 | 0 |  |
|  | Tf_00095 | 334 |  | marine_69981 | 97.6 | 334 | 0 |  |
|  | Tf_00095 | 334 |  | marine_114812 | 96.8 | 281 | 0 |  |
|  | Tf_00095 | 334 |  | marine_112795 | 98.9 | 273 | 0 |  |
|  | Tf_00095 | 334 |  | marine_88969 | 100 | 220 | 1.00E-157 |  |
|  | Tf_00095 | 334 |  | marine_80564 | 97.62 | 84 | 1.00E-54 |  |
|  | T3f_00206 | 358 | Glyceraldehyde-3-phosphate dehydrogenase 3 | marine_62105 | 82.65 | 340 | 0 | Reductive pentose phosphate cycle |
|  | Tf_03342 | 78 | Phosphoenolpyruvate synthase | marine_85820 | 98.72 | 78 | 7.00E-50 | Reductive citrate cycle |
| Pelagibacterales | Tf_01232 | 146 | Succinate dehydrogenase flavoprotein subunit | marine_29437 | 100 | 110 | 8.00E-82 |  |
|  | Tf_01232 | 146 | Succinate dehydrogenase flavoprotein subunit | marine_30722 | 100 | 87 | 2.00E-59 |  |
|  | Tf_01232 | 146 | Succinate dehydrogenase flavoprotein subunit | marine_04977 | 100 | 76 | 2.00E-55 |  |
|  | Tf_01232 | 146 | Succinate dehydrogenase flavoprotein subunit | marine_34085 | 97.37 | 38 | 2.00E-21 |  |
|  | Tf_01405 | 48 | Pyruvate, phosphate dikinase | marine_31386 | 100 | 48 | 1.00E-31 |  |
|  | Tf_01405 | 48 | Pyruvate, phosphate dikinase | marine_23017 | 100 | 48 | 2.00E-31 |  |
|  | Tf_01405 | 48 | Pyruvate, phosphate dikinase | marine_31178 | 97.92 | 48 | 8.00E-31 |  |
|  | Tf_01405 | 48 | Pyruvate, phosphate dikinase | marine_26749 | 97.83 | 46 | 2.00E-29 |  |
|  | Tf_01405 | 48 | Pyruvate, phosphate dikinase | marine_39164 | 97.83 | 46 | 4.00E-29 |  |
|  | Tf_01405 | 48 | Pyruvate, phosphate dikinase | marine_07613 | 100 | 45 | 5.00E-29 |  |
|  | Tf_01405 | 48 | Pyruvate, phosphate dikinase | marine_28296 | 97.83 | 46 | 5.00E-29 |  |
|  | Tf_01405 | 48 | Pyruvate, phosphate dikinase | marine_40988 | 100 | 44 | 5.00E-28 |  |
|  | T3f_00329 | 164 | All-trans-phytoene synthase | marine_04783 | 78.66 | 164 | 1.00E-88 | β-carotene biosynthesis |
|  | Tf_00330 | 102 | Squalene/phytoene synthase | marine_30352 | 50.98 | 102 | 2.00E-31 | β-carotene biosynthesis |
|  | Tf_00331 | 485 | Phytoene desaturase (lycopene-forming) | marine_04782 | 90.1 | 485 | 0 | β-carotene biosynthesis |
|  | Tf_00389 | 313 | Fructose-1,6-bisphosphatase class 2 | marine_03767 | 86.5 | 311 | 0 | Reductive citrate cycle |
