# Supplementary figures and images for: Raman‐activated cell sorting and metagenomic sequencing revealing carbon‐fixing bacteria in the ocean
Source: Environ Microbiol. 2018 Jul 2;20(6):2241–55. doi: 10.1111/1462-2920.14268 (PMC6849569; doi:10.1111/1462-2920.14268)

Fig. S1

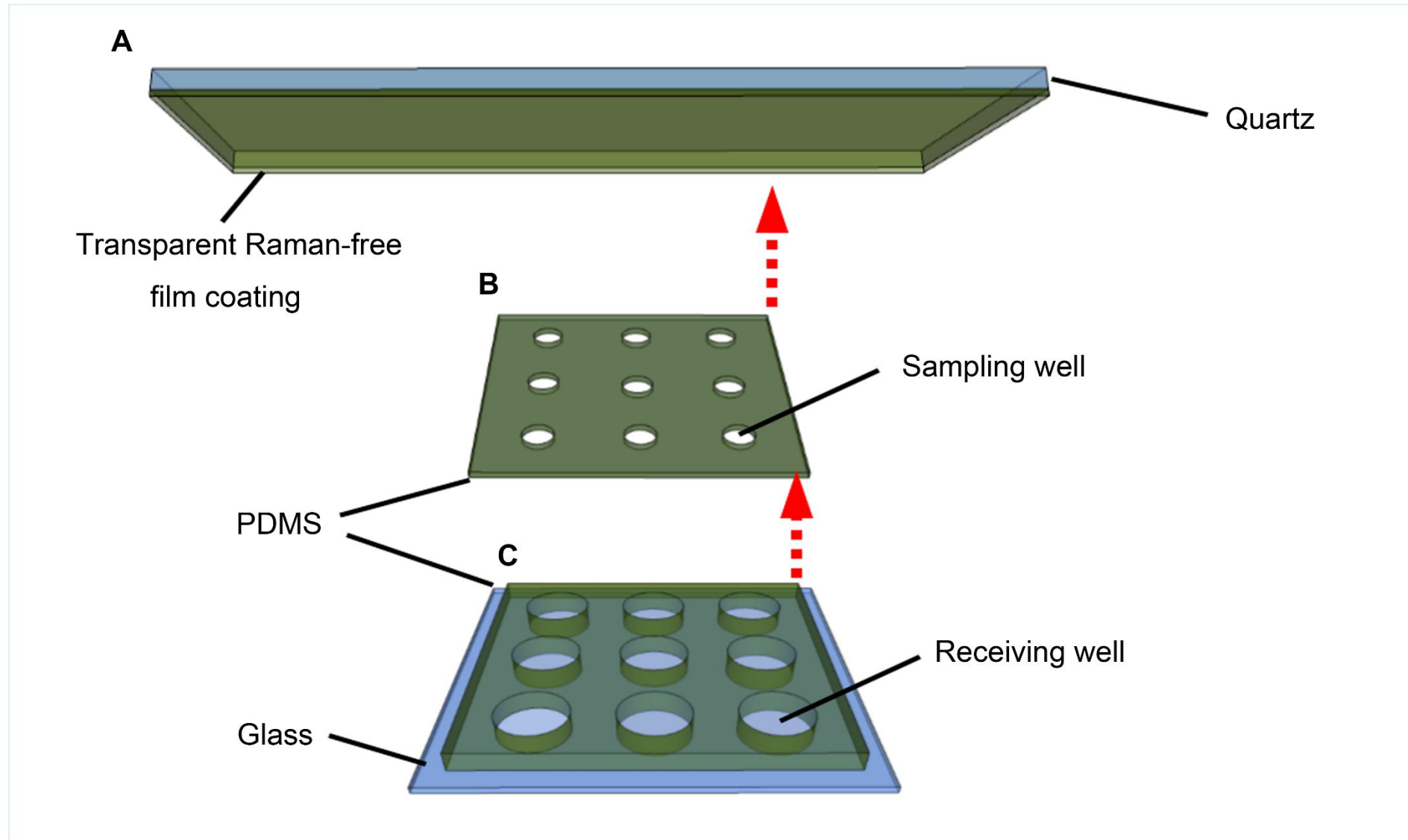

Fig. S2

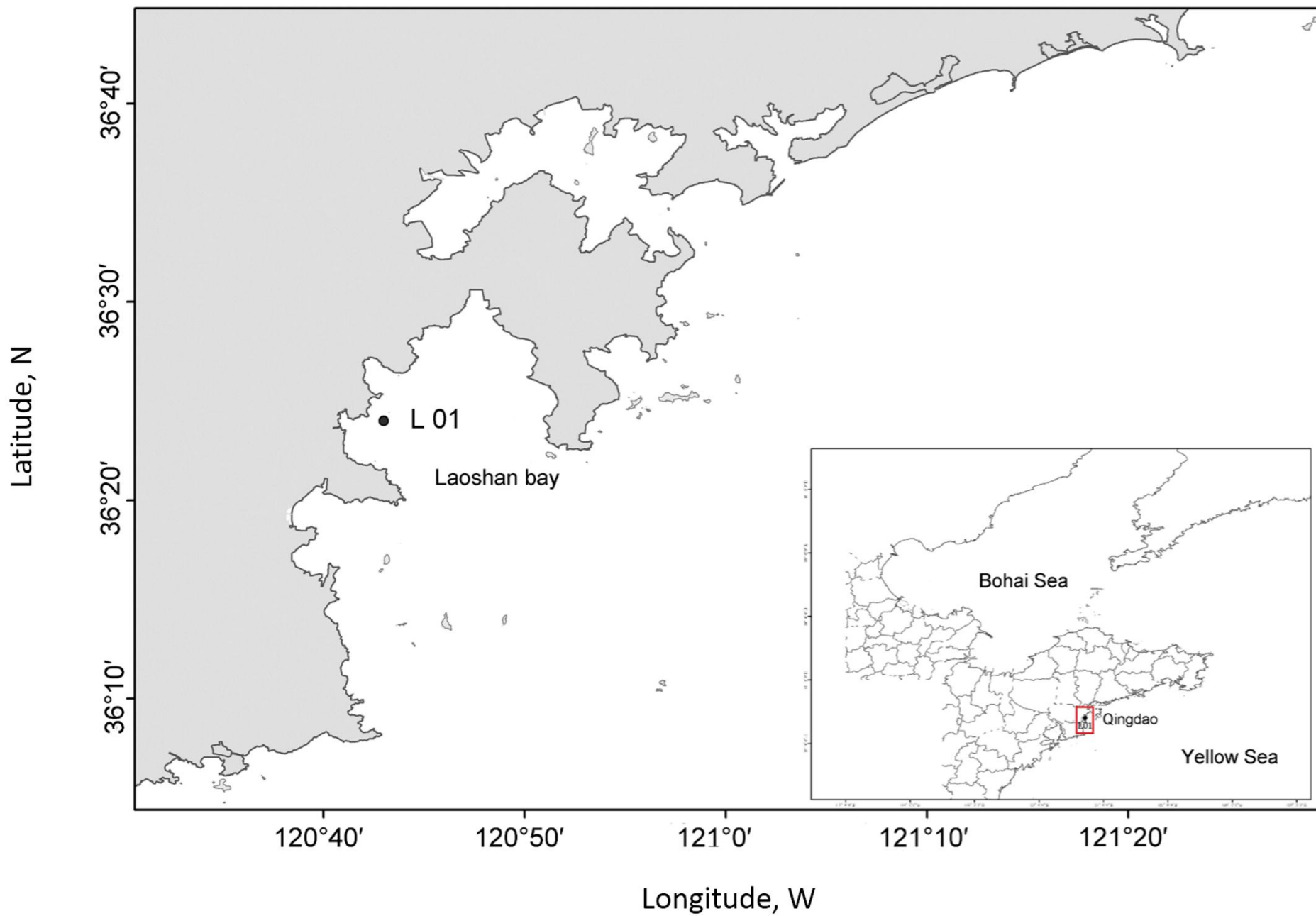

Fig. S3

**A**

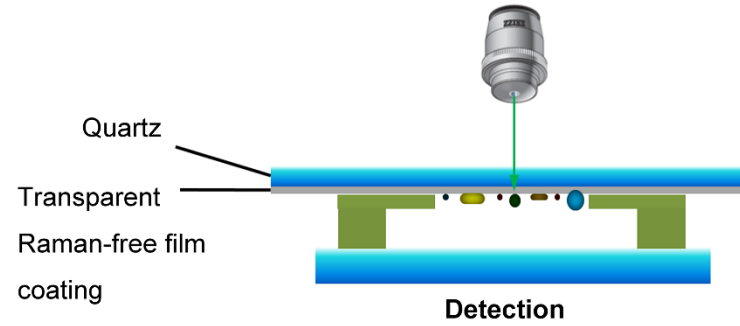

**B**

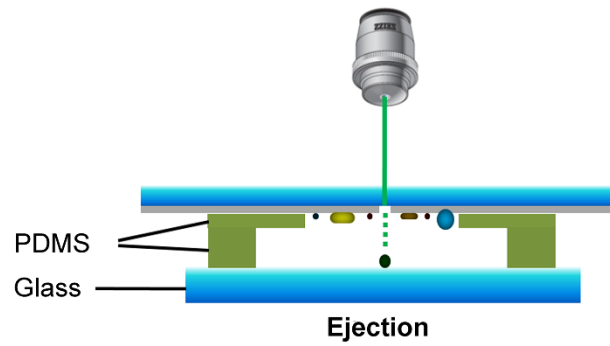

**C**

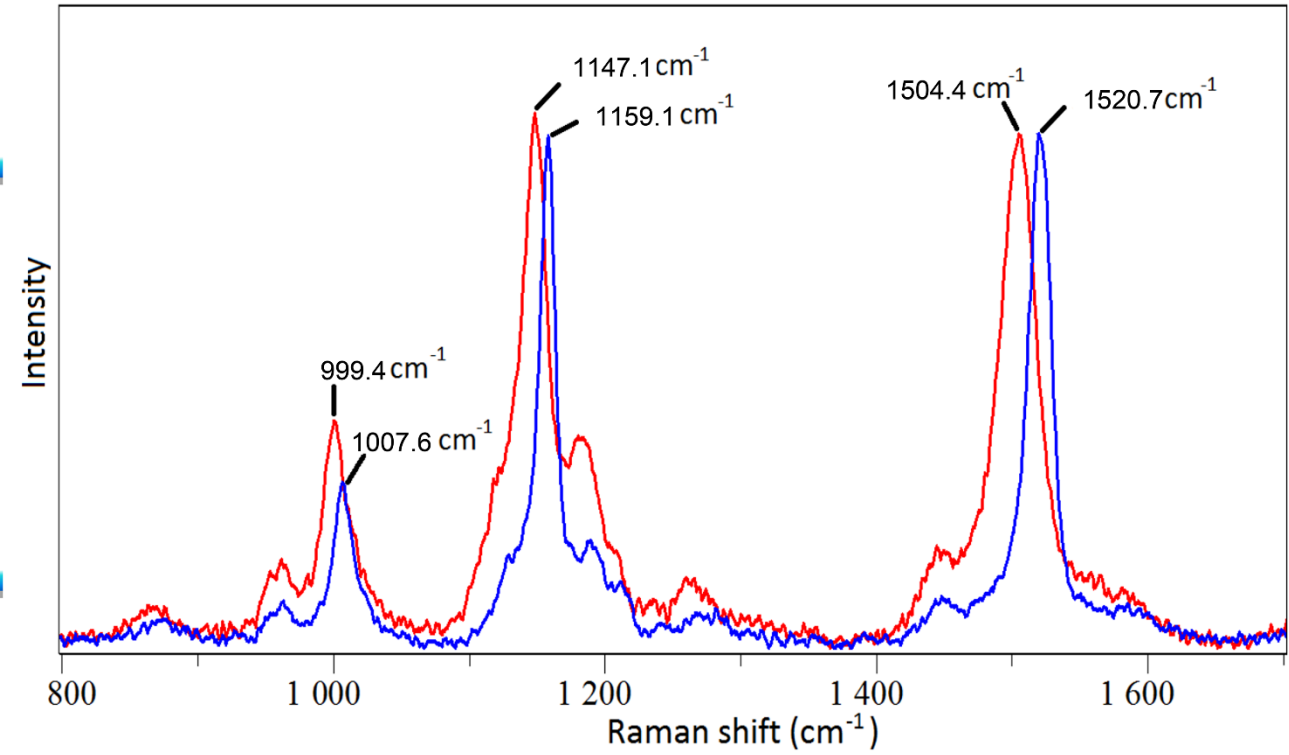

Fig. S4

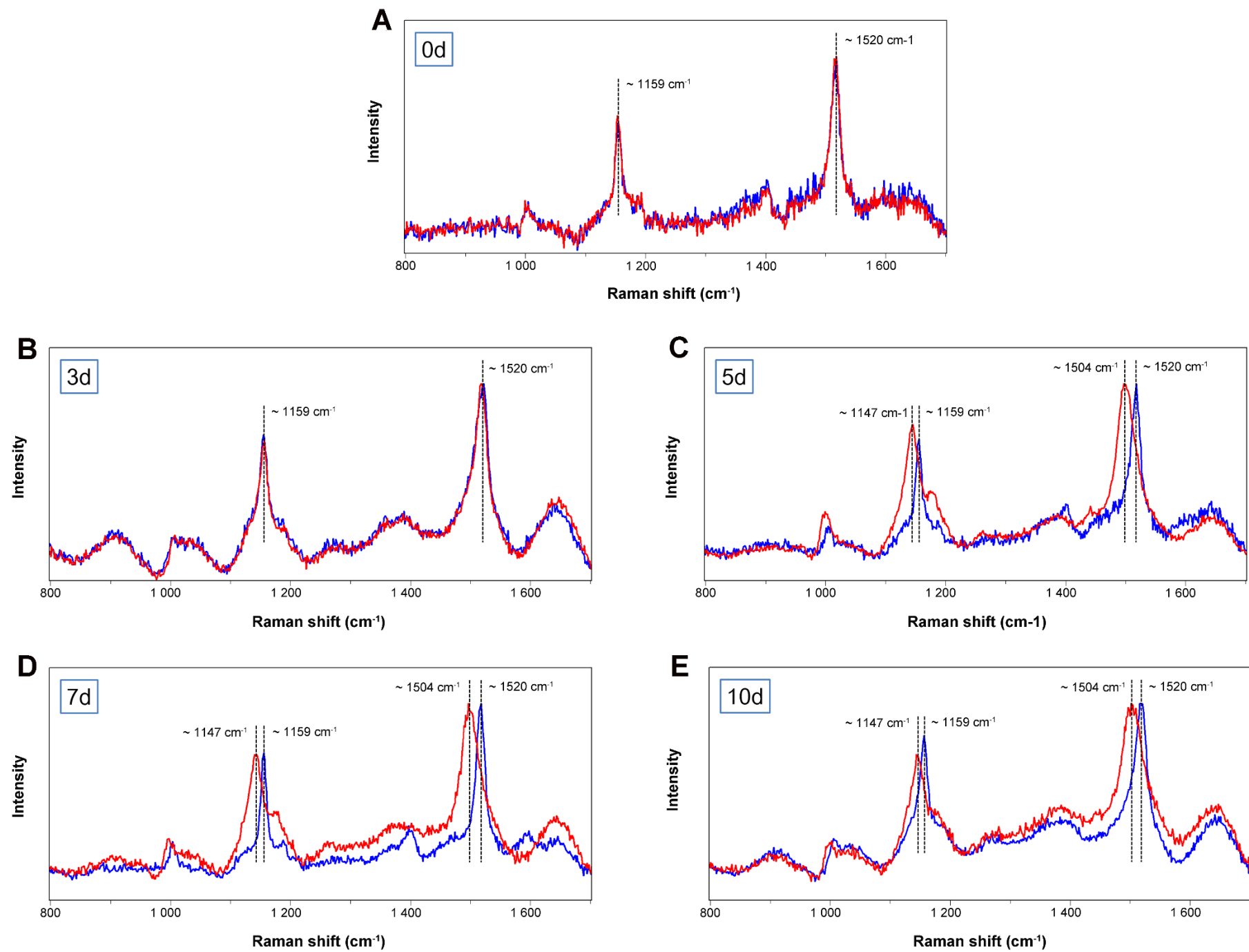

Fig. S5

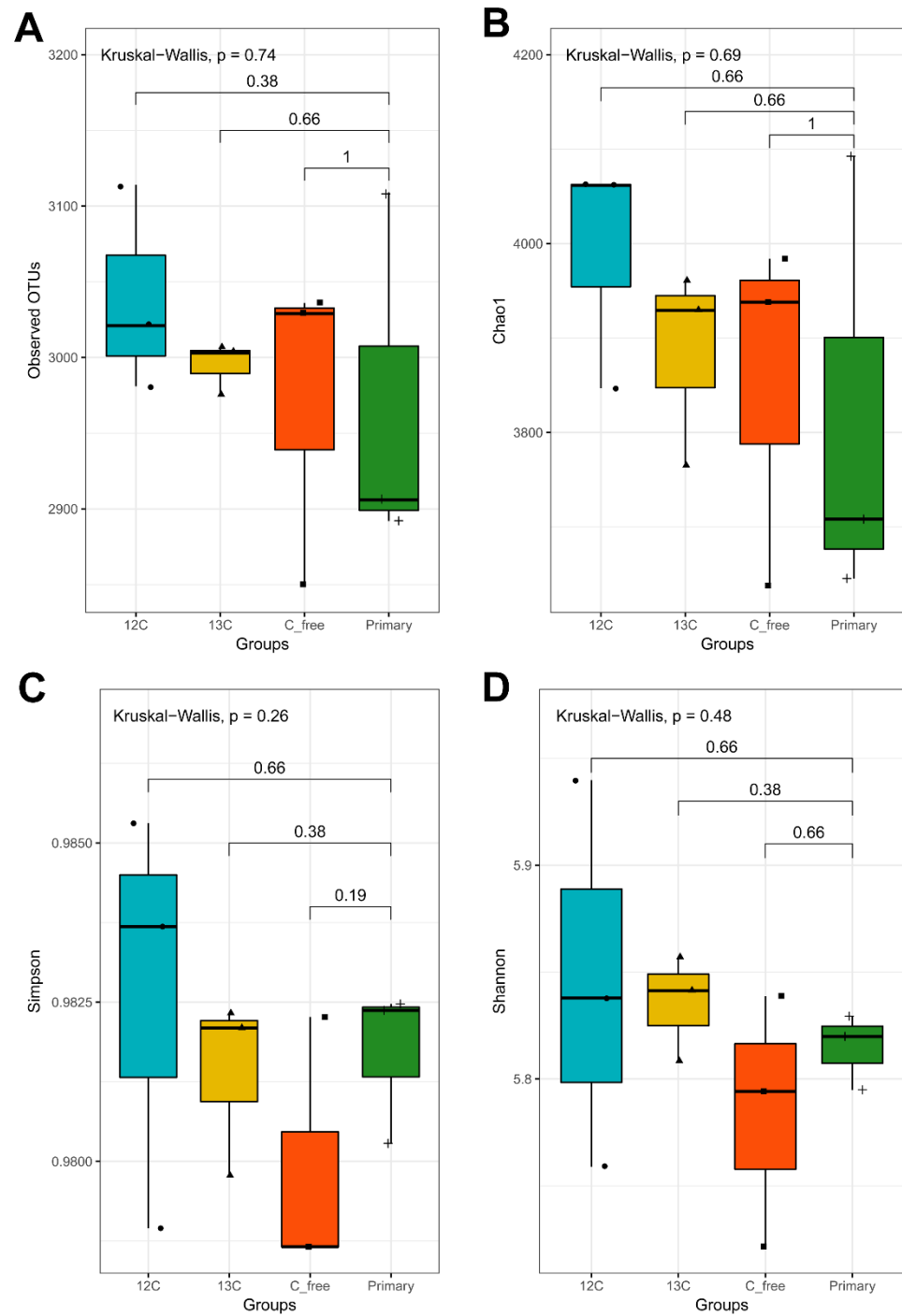

Fig. S6

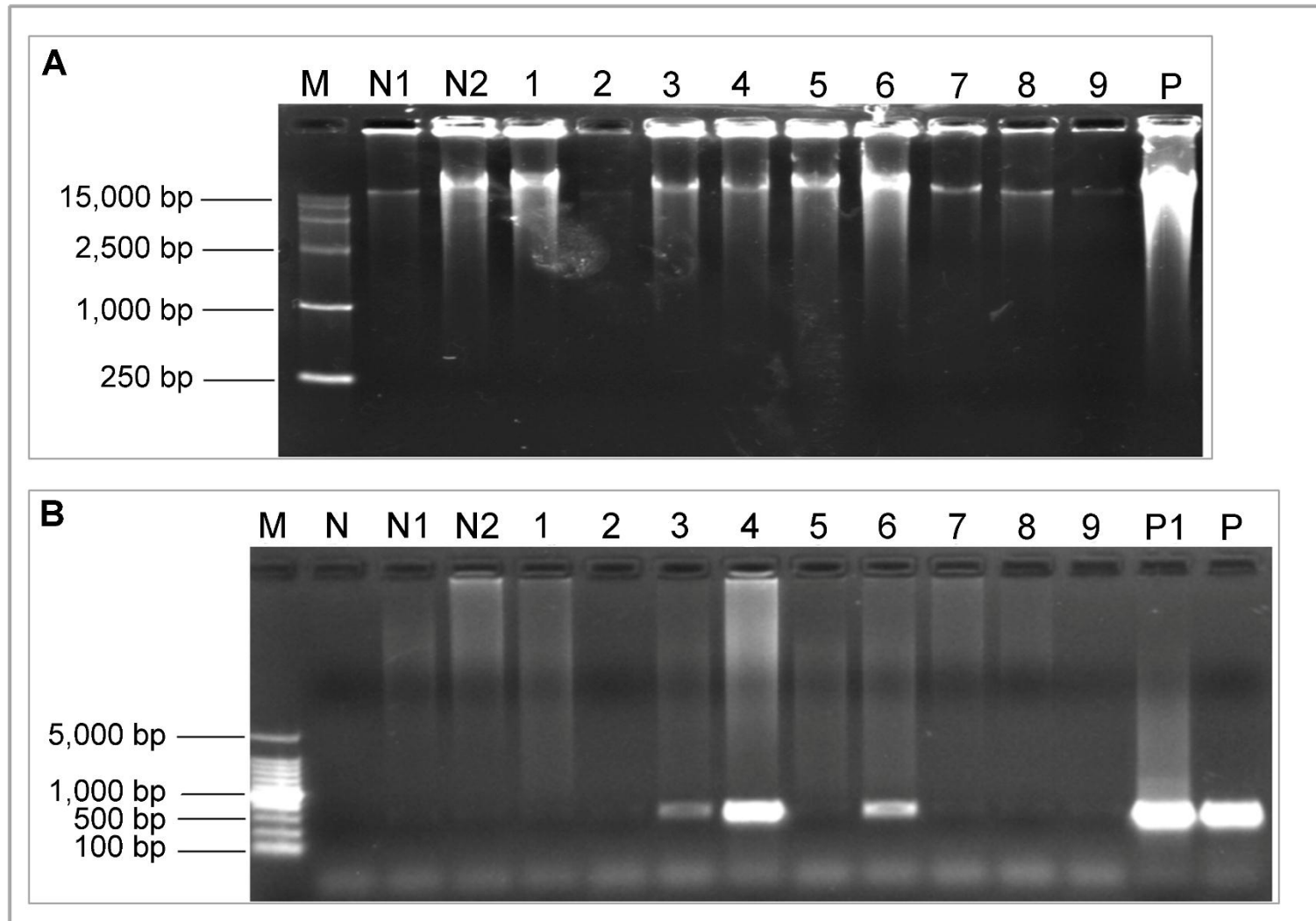

Fig. S7

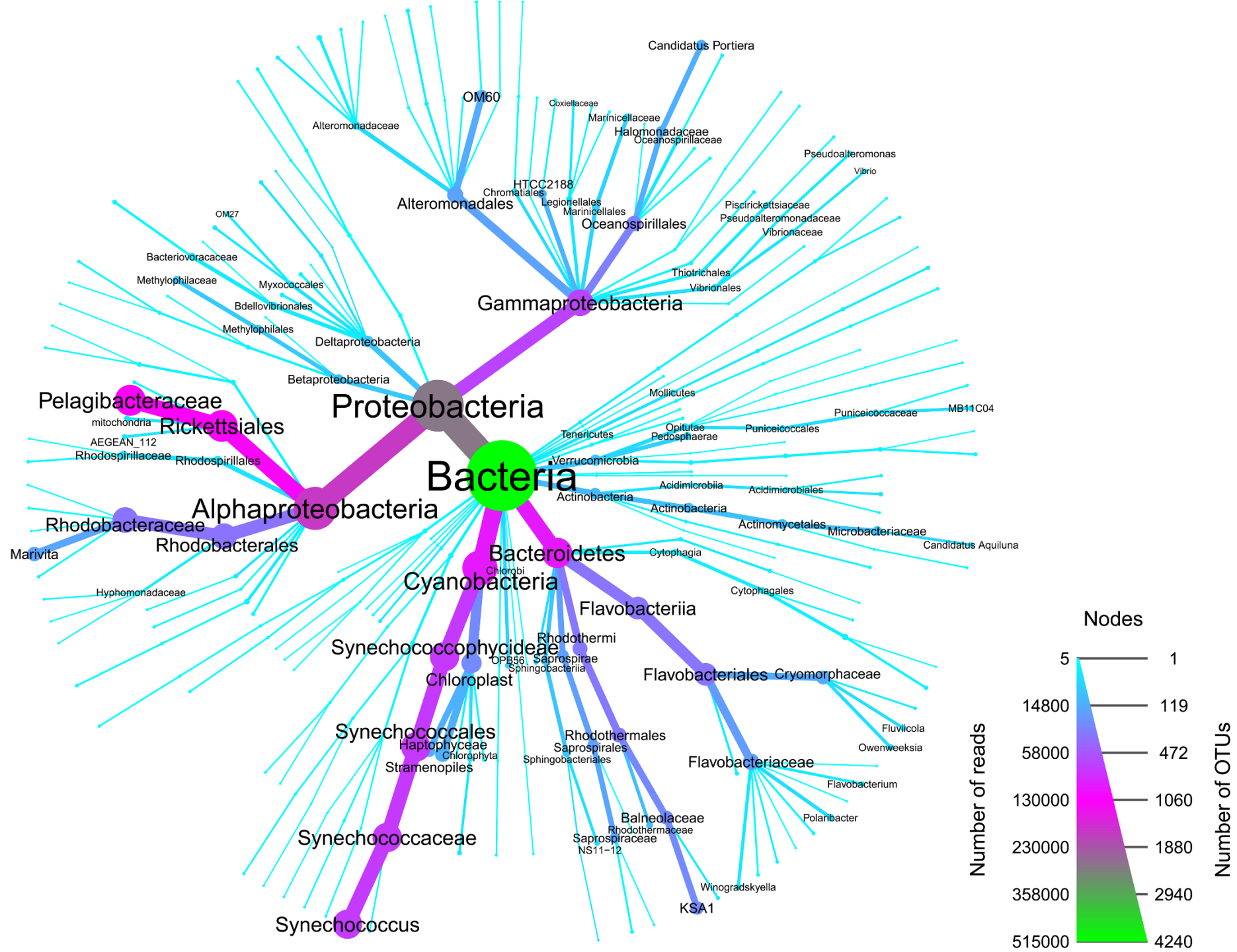

Fig. S8

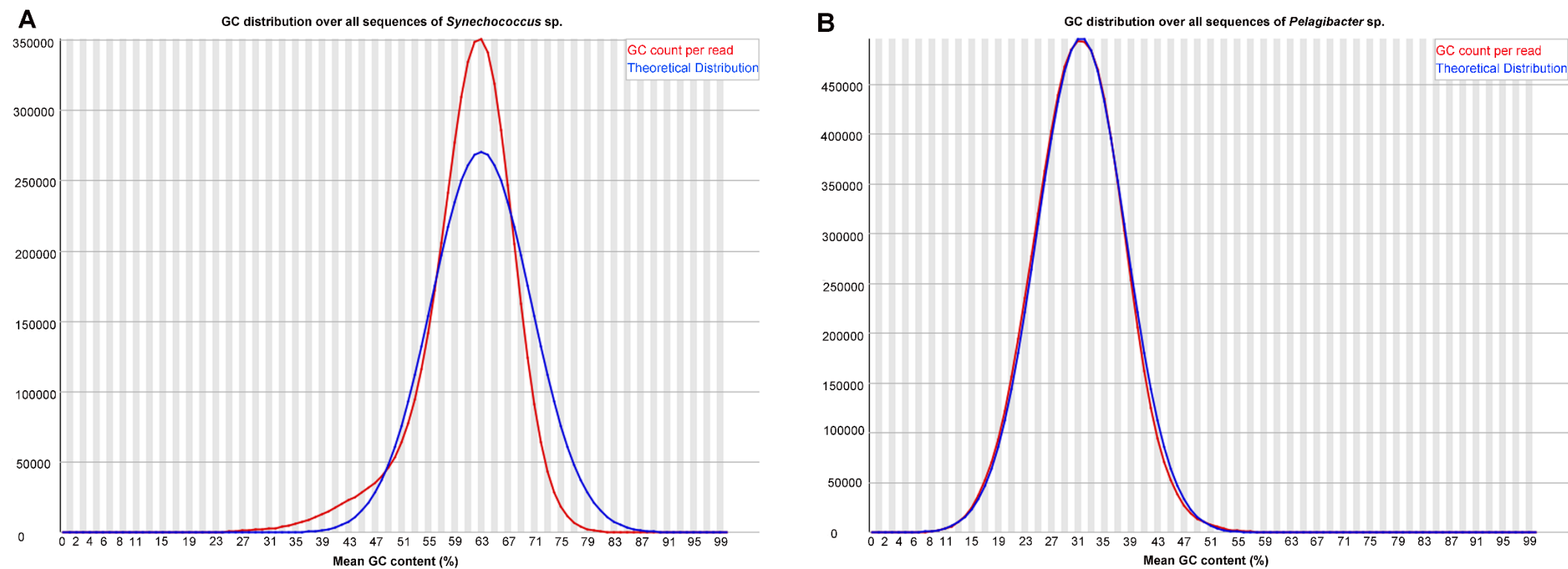

Fig. S9

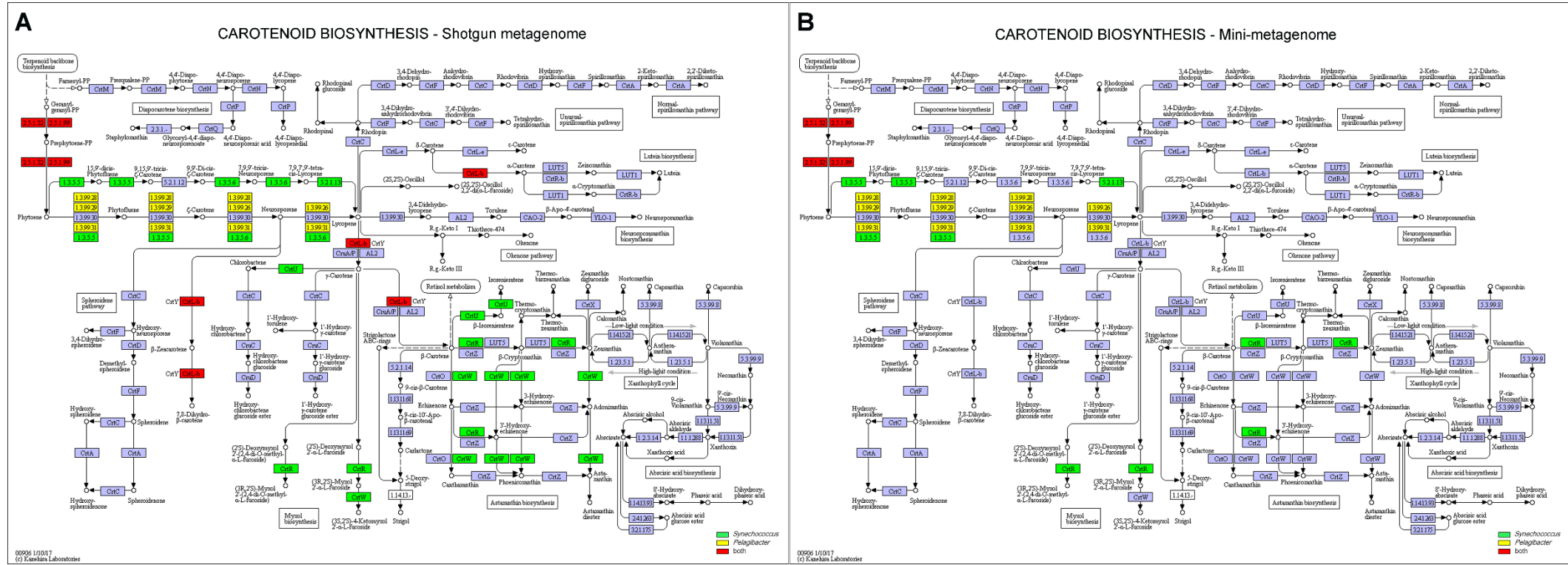

Fig. S10

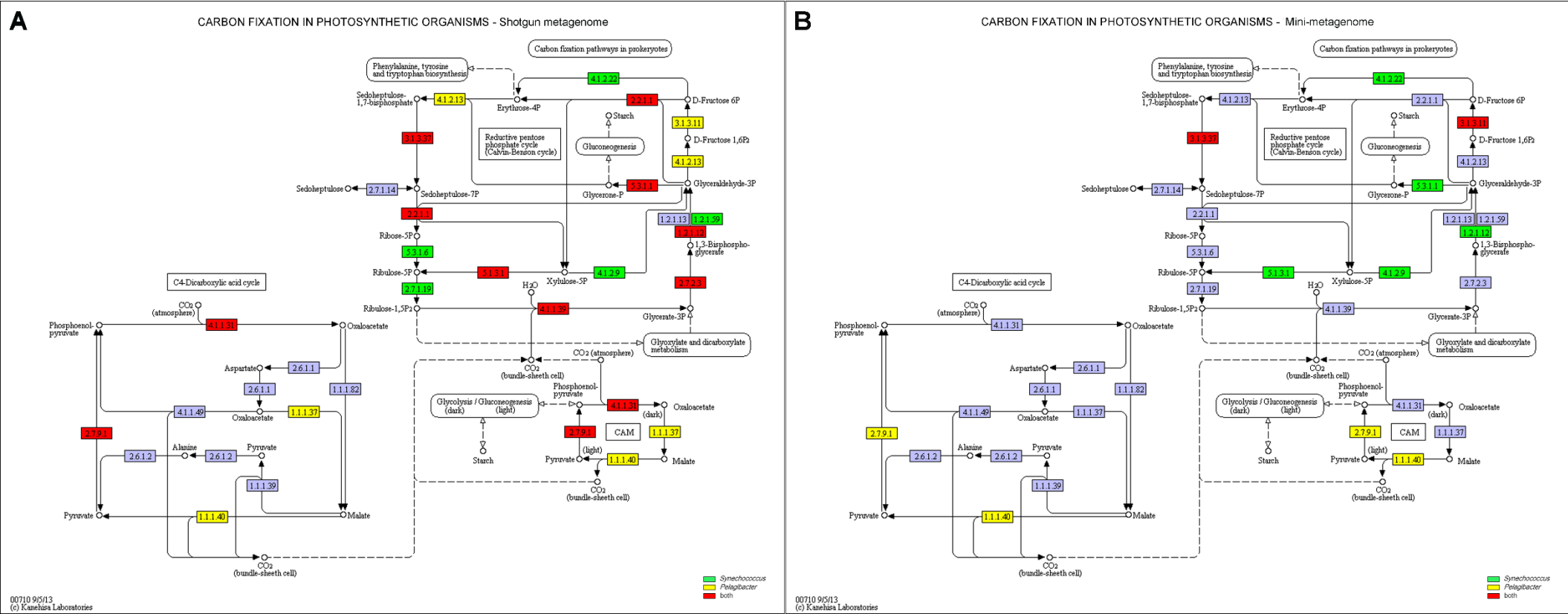

Fig. S11

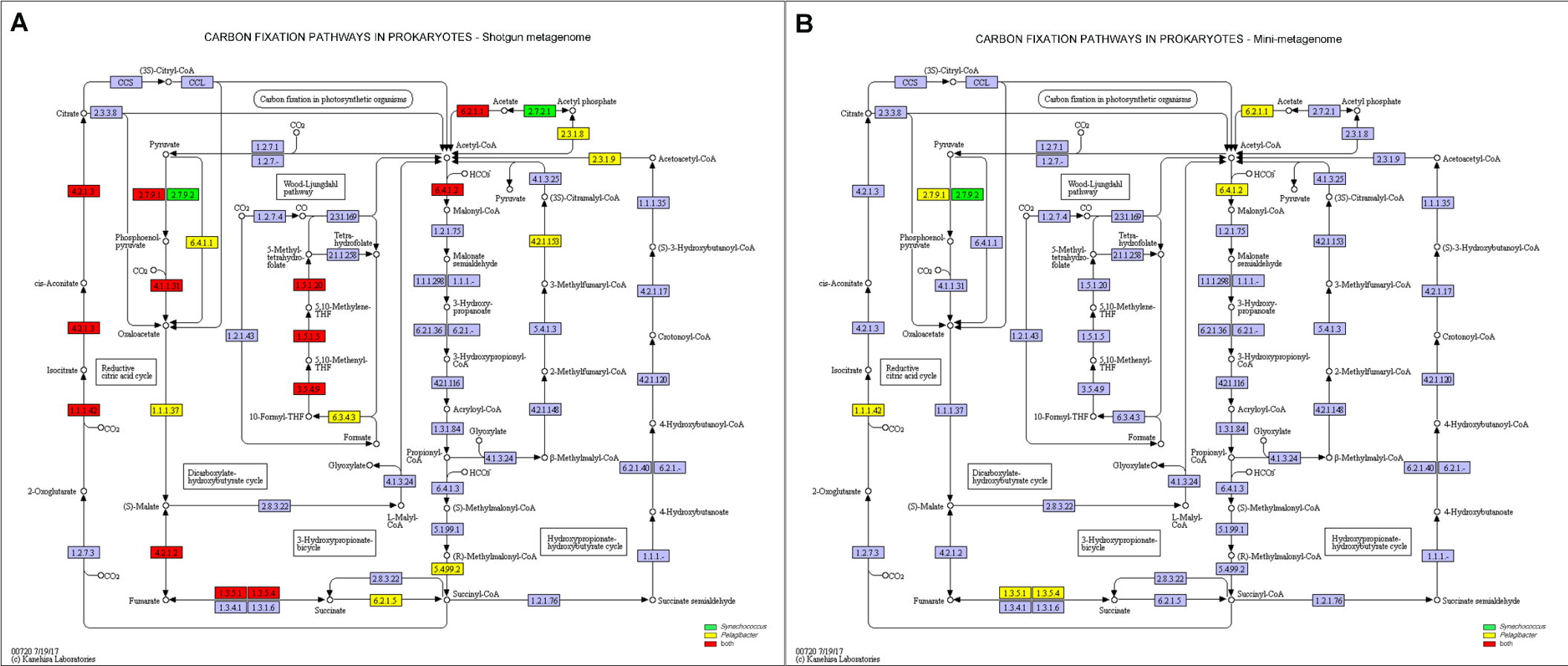

Fig. S12

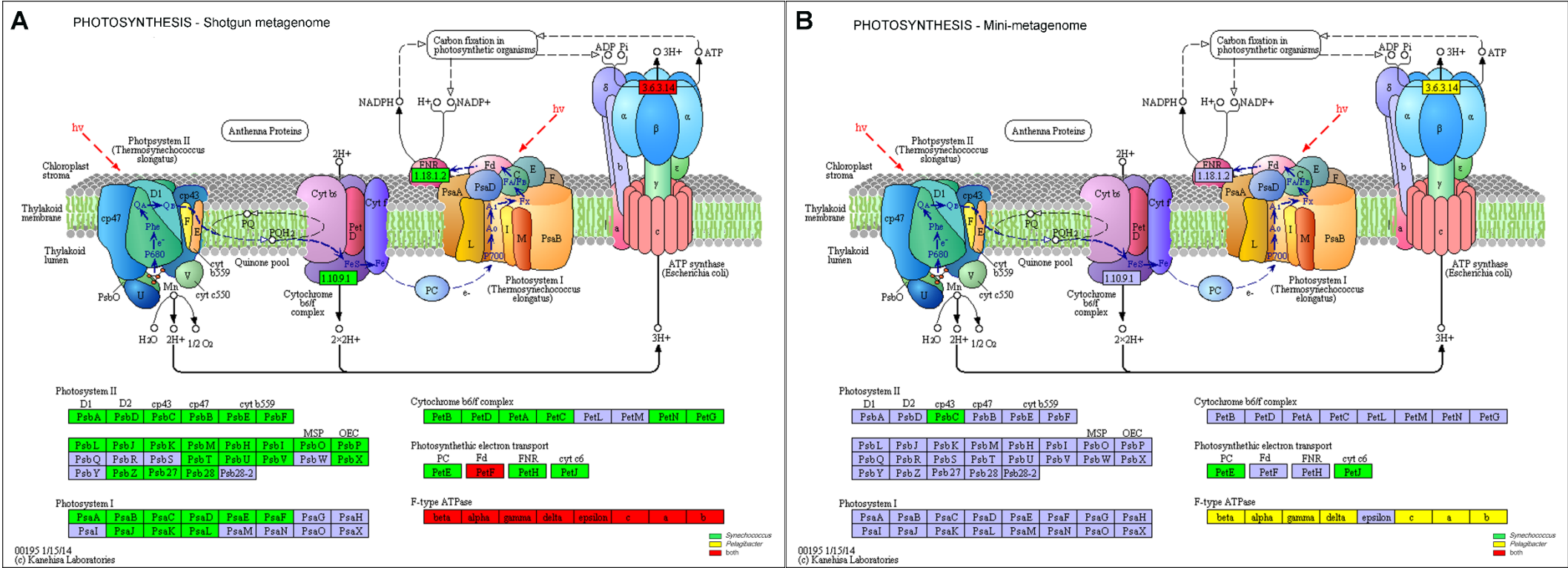

Fig. S13

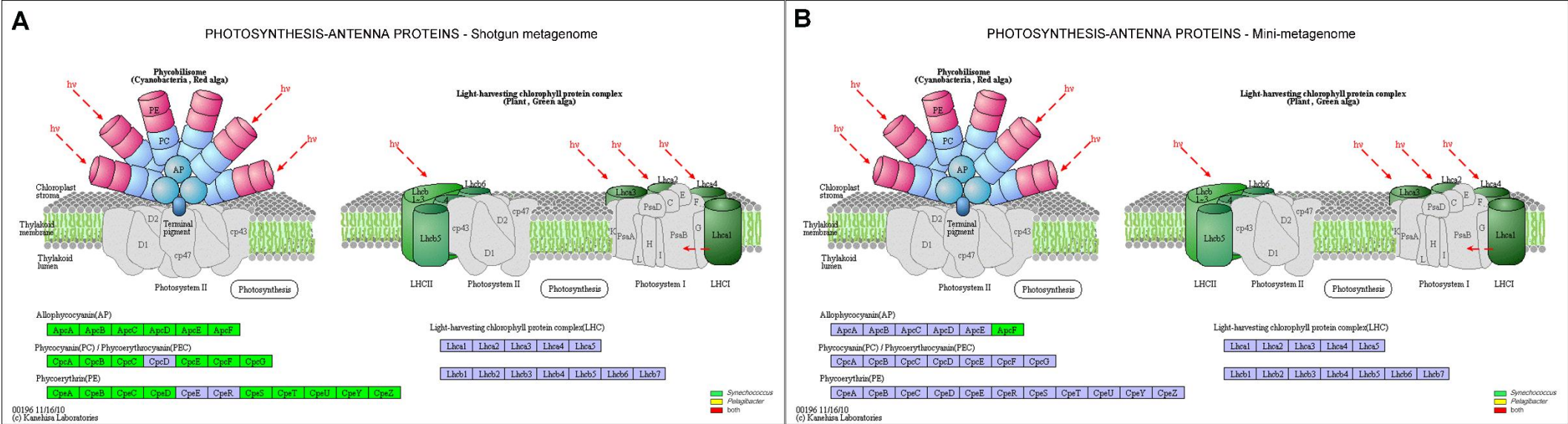

Fig. S14

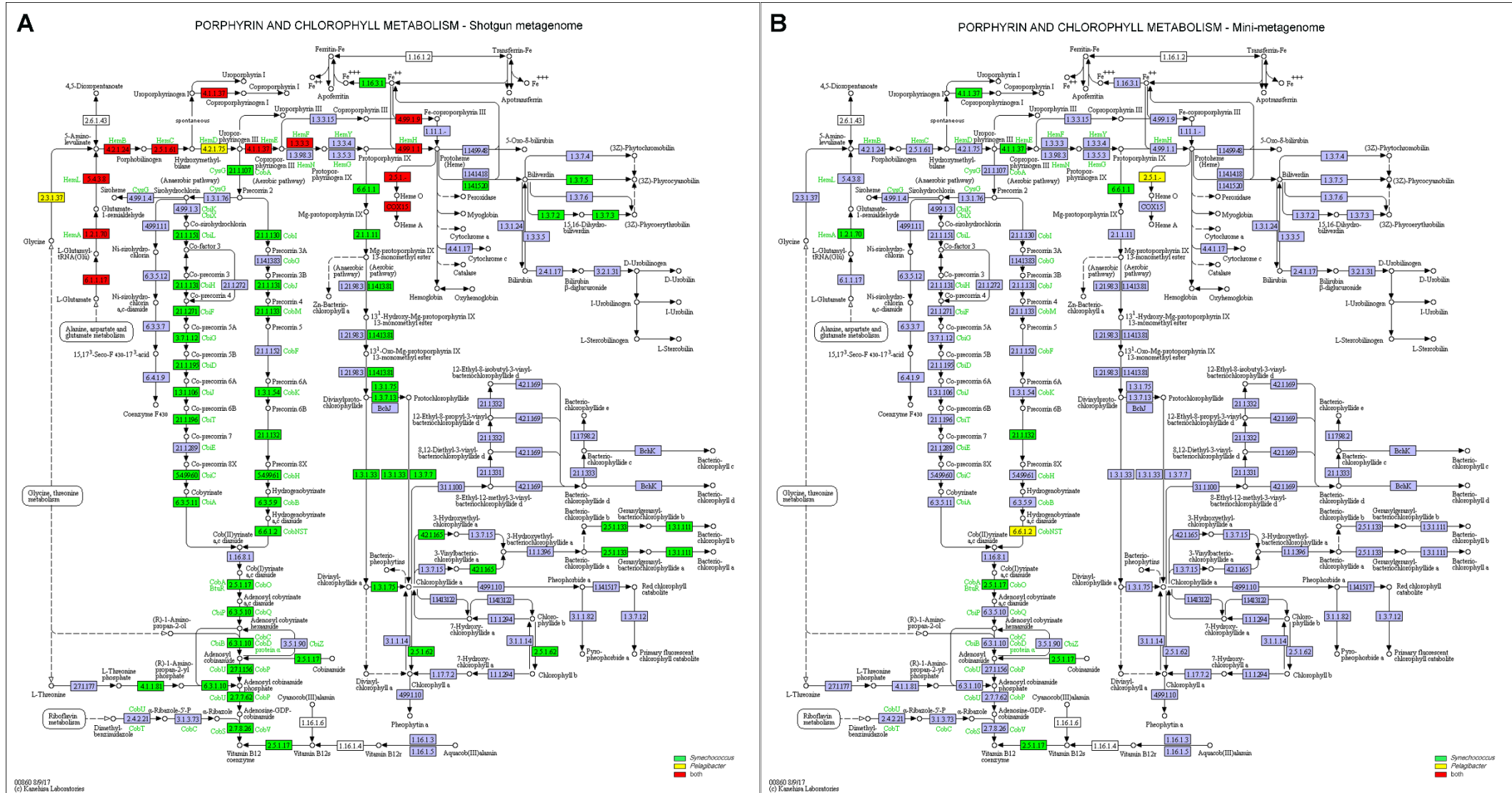

Fig. S15

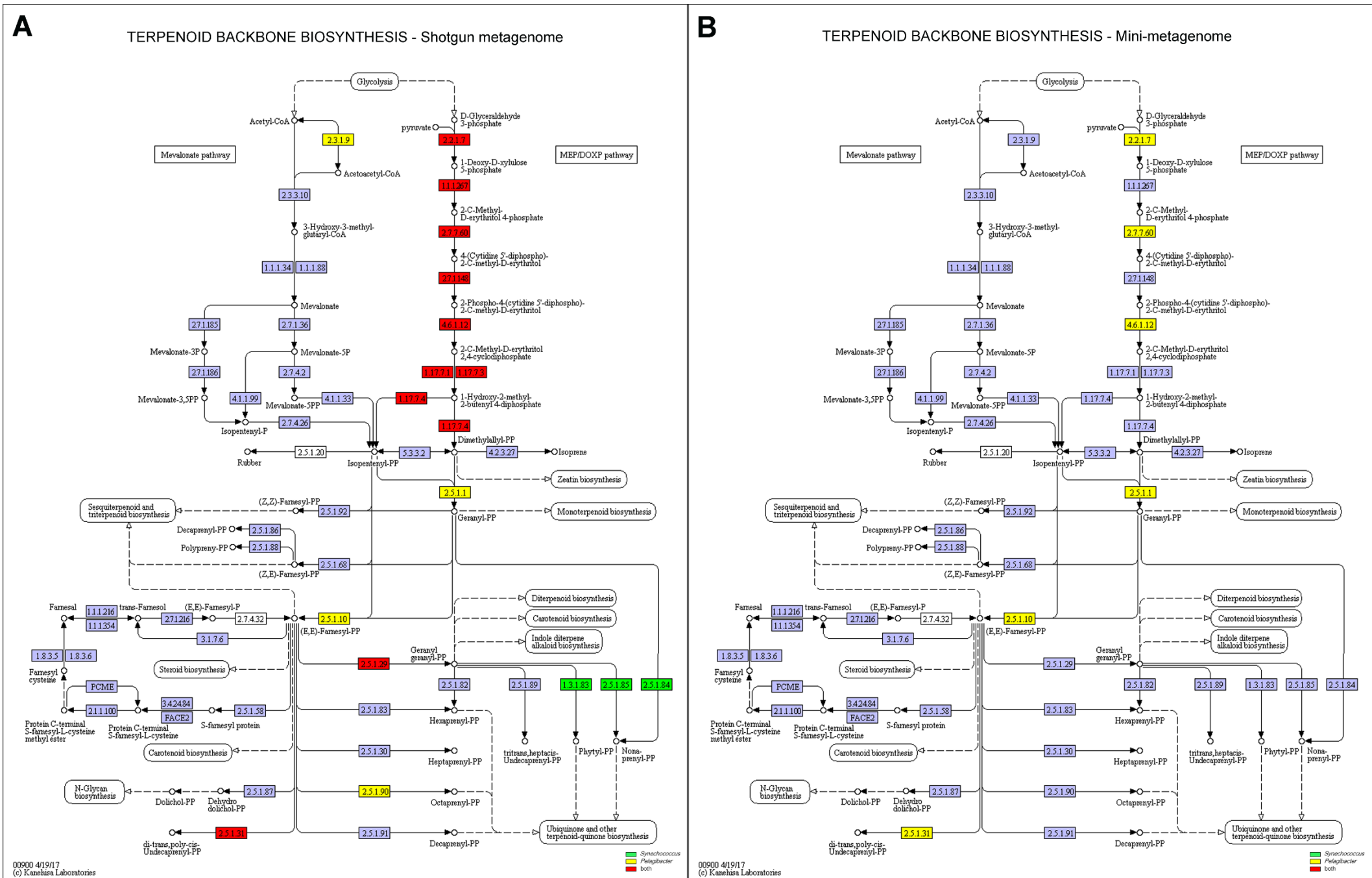

Supplement: Supplementary file 2 — Fig. S1. Illustration of the ‘all‐in‐one’ device assembly. A. Transparent ejection slide. B. Sampling chip. C. Cell collection chip. Fig. S2. Geographic location of sampling site in Yellow Sea. The label ‘L 01’ represents the location position of sampling site. Fig. S3. Scheme of Raman identification and sorting of CO2‐fixing microbes with ‘all‐in‐one’ integrated device. A. Acquisition of single cell Raman spectra and CO2‐fixing microbes identification. B. Sorting cells of interest by laser ejection. C. Shifts of the carotenoid Raman bands in single cell Raman spectra of Synechococcus spp. PCC7002, indicating 13C incorporation into the cell when cells were incubated with 13C NaHCO3. Fig. S4. Raman spectra of carotenoids containing cells in the seawater which were incubated in closed bottles at room temperature at different times. A. The average Raman spectra of cells treated with 13C NaHCO3 and 12C NaHCO3, respectively, at time t = 0 days. B. The average Raman spectra of cells treated with 13C NaHCO3 and 12C NaHCO3, respectively, at time t = 3 days. C. The average Raman spectra of cells treated with 13C NaHCO3 and 12C NaHCO3, respectively, at time t = 5 days. D. The average Raman spectra of cells treated with 13C NaHCO3 and 12C NaHCO3, respectively, at time t = 7 days. E. The average Raman spectra of cells treated with 13C NaHCO3 and 12C NaHCO3, respectively, at time t = 10 days. Fig. S5. Alpha diversity comparisons across four different treatments of the seawater: red bars – 12C NaHCO3 amended sample (12C); blue bars – 13C NaHCO3 amended sample (13C); green bars – control sample without NaHCO3 (C_free); purple bars – original seawater sample control (primary). A. Box plot showing the variation of observed OTUs. B. Box plot showing the variation of Chao1 index. C. Box plot showing the variation of Simpson index. D. Box plot showing the variation of Shannon index. Fig. S6. Agarose gel images of the multiple displacement amplifications (MDAs) and 16S rRNA gene validat [file EMI-20-2241-s002.pdf]
