# Supplementary material for: Raman‐activated cell sorting and metagenomic sequencing revealing carbon‐fixing bacteria in the ocean
Source: Environ Microbiol. 2018 Jul 2;20(6):2241–55. doi: 10.1111/1462-2920.14268 (PMC6849569; doi:10.1111/1462-2920.14268)
Supplement: Supplementary file 5 — File S3. Identified proteorhodopsin (PR) genes from metagenomic bins of Pelagibacter spp. [file EMI-20-2241-s005.docx]

**Supplementary file 3. Identified proteorhodopsin (PR) genes from metagenomic bins of *Pelagibacter* spp.**

>marine_00065 Green-light absorbing proteorhodopsin precursor

ATGGATTTAATTTCCCTTGACGCTTCGCAGTACTCGCTGGTGTATAACATGTTCTCATTCACAATTGCATCGATGCTAGCTGCGTTTGTGTTTTTCTGGATGGGTCAGCAACAAGTTGCACCGAAGTACCGTATTTCCTTGATTGTCTCTGGATTGGTTGTAGGTATTGCTGCTTACCACTACTTCAGAATCTTTAGCAGCTTCGAGCATGCTTACGACGCTGCTACTGGAATGTTCGACACCGCTGGATTCAACGACGCTTACCGCTACGTTGACTGGCTACTGACTGTACCACTCCTGTTGGTCGAGCTAGTACTTGTATTAGGTCTTTCAAAAGAGAAGAATGCAGCCCTACTTCCAAAGCTTGTCATCGCATCTGCCTTTATGATCGCGCTTGGATATCCAGGTGAAATCATGGCGTTTGGTGACCGTGAGCTCTTTGGAGCATACGGCCTGTGGGGTACACTCTCTACGATTCCATTTGTTTACATTCTATTCGTTCTTTGGGGCGAGTTAGGGAAAGCAATGGAAGATCAACCAGACAATGTTCGCGTACTTTTCCGCAACATTCGTCTTCTATTGATCGGTACATGGGGTTTCTACCCAATCGTATACTGTCTACCATTCTTCGGTATTGAGCAAGGAAACACACTTGTTGCAATCCAAGTTGGTTATGCATTGGCTGATGTATTAGCGAAAGCTGGATACGGAGTCATGATCTATGCGATTGCTCGTGCGAAGTCTGAAGCAGCTGGATTTAAGCTTGCTGAAGCCTAA

>marine_05527 Blue-light absorbing proteorhodopsin precursor

GTACCGTTATTGATGTTAGAATTCTATTTTGTTCTATCAGCAGTAAACAAAGCAGACTCTGGAATTTTCTGGAGACTGATGATTGGTACATTAGTAATGCTAGTAGGTGGATACTTAGGAGAAGCAGGATACATCAACGTCACACTTGGTTTCATAATCGGTATGGCTGGTTGGGTATACATTCTTTATGAAGTATTCTCTGGTGAAGCAGGTAAGAGAGCAGCGAAAAGTGGTAATAAAGCACTCGTAACTGCTTTTGGTGCAATGAGAATGATCGTTACTGTAGGTTGGGCTATTTACCCGTTAGGTTACATTTTTGGTTACATGACAGGTGGAGTAGACGCTAGCTCACTTAACGTGATTTACAACGCAGCTGACTTCTTGAACAAGATCGCTTTCGGTCTAATCATTTGGGCAGCAGCAATGCAACAACCTGGTAGAGCTAAGTAA

>marine_07492 Blue-light absorbing proteorhodopsin precursor

GTACCGTTATTGATGTTAGAATTCTATTTTGTTCTATCAGCAGTAAACAAAGCAGACTCTGGAATTTTCTGGAGACTGATGATTGGTACATTAGTAATGCTAGTAGGTGGATACTTAGGAGAAGCAGGATACATCAACACTACACTTGGTTTCATTATCGGTATGGCTGGTTGGGTATACATTCTTTATGAAGTATTCTCTGGTGAAGCAGGTAAGAGAGCAGCGAAAAGTGGTAACAAAGCACTTGTAACTGCTTTTGGTGCAATGAGAATGATCGTTACAGTAGGTTGGGCTATTTACCCGTTAGGTTACATTTTTGGTTACATGACAGGTGGAGTAGACGCTAGCTCACTAAACGTGATTTACAACGCAGCTGACTTCTTGAACAAGATCGCTTTCGGTCTGATCATTTGGGCAGCAGCAATGCAACAACCTGGTAGAGCTAAGTAA

>marine_12216 Blue-light absorbing proteorhodopsin precursor

AACTTAGGTTTCATCATTGGTATGGCTGGATGGATTTACATCCTGTATGAAGTATTTGCTGGTGAAGCAGGTCAAGAAGCGAAAAAATTAAAAGGCGCTGCAGCGAGCTGTTTCGAATTCTGTAAATGGATCGTAACAATCGGTTGGGCAGTATATCCATTAGGTTACCTATTTGGATATATGACAGGTTCAGCAGATGAAGTTTCATTAAACATCATTTACAACCTAGCAGACTTTATTAACAAGTTGATCTTTGGTTTAGTGATCTGGCATACAGCTAAGTCACTTACTCCTCAGACAGCGAAGTAA

>marine_13198 Blue-light absorbing proteorhodopsin precursor

GCTACTGGTGACTCACCAACTGTTTACAGATACATTGACTGGTTAATTACTGTACCGTTATTGATGTTAGAATTCTATTTTGTTCTATCAGCAGTAAACAAAGCAGACTCTGGAATTTTCTGGAGACTGATGATTGGTACATTAGTAATGCTAGTAGGTGGATACTTAGGAGAAGCAGGATACATCAACGCTACACTTGGTTTCATTATCGGTATGGCTGGTTGGGTATACATTCTTTATGAAGTATTCTCAGGTGAAGCAGGTAAGAGAGCAGCGAAAAGTGGTAATAAAGCACTTGTAACTGCTTTTGGTGCAATGAGAATGATCGTTACTGTAGGTTGGGCTATTTACCCATTAGGTTACATTTTTGGTTACCTAACAGGTGGAGTAGACGCTAACTCACTTAACGTGATTTACAACGCAGCTGACTTCTTGAACAAGATCGCTTTCGGTCTGATCATTTGGGCAGCA

>marine_13635 Blue-light absorbing proteorhodopsin precursor

GGTATGGCTGGTTGGGTATACATTCTTTATGAAGTATTCTCAGGTGAAGCAGGTAAAAGAGCAGCGAAAAGTGGTAATAAAGCACTTGTAACTGCTTTTGGTGCAATGAGAATGATCGTTACTGTAGGTTGGGCTATTTACCCATTAGGTTACATTTTTGGTTACCTAACAGGTGGAGTAGATGCTAACTCACTTAACGTGATTTACAATGCAGCTGACTTTTTGAACAAGATCGCTTTTGGTCTAATCATTTGGGCAGCAGCTATGCAACAACCTGGTAGAGCTAGATAA

>marine_14512 Blue-light absorbing proteorhodopsin precursor

CCGTTAGGTTACATTTTTGGTTACATGACAGGTGGAGTAGACGCTAGCTCACTAAACGTGATTTACAACGCAGCTGACTTCTTGAACAAGATCGCTTTCGGTCTGATCATTTGGGCAGCAGCGATGCAACAACCTGGTAGAGCTAAGTAA

>marine_15633 Blue-light absorbing proteorhodopsin precursor

GTATACATTCTTTATGAAGTATTCTCTGGTGAAGCAGGTAAGAGAGCAGCGAAAAGTGGTAACAAAGCACTTGTAACTGCTTTTGGTGCAATGAGAATGATCGTTACAGTAGGTTGGGCTATTTACCCGTTAGGTTACATCTTTGGTTACATGACAGGTGGAGTAGACGCTAGCTCACTAAACGTGATTTACAACGCAGCTGACTTCTTGAACAAGATCGCTTTCGGTCTGATCATTTGGGCAGCAGCAATGCAACAACCTGGTAGAGCTAAGTAG

>marine_19797 Green-light absorbing proteorhodopsin precursor

GTTGATTGGGTATTAACTGTGCCTTTAATGTGTGTAGAATTCTACCTCATTTTAAAAGTTGCAGGAGCCAAAAAATCTTTAATGTGGAAACTAATTGGTCTATCGGCAATTATGTTAGTGACAGGATACTTCGGAGAAGCAGTCTACAGAGATCAGGCATGGTTCTGGGGCTTAGTATCAGGTATCGCTTATTTCATTATCGTTTACATGATATGGCTTGGGGAAGCGAAGAAATTGGCAGAAGAAGCAGGAGGTGCAGTACTTAAGGCCCACAAAACGTTATGCTGGTTTGTACTTGTTGGTTGG

>marine_23415 Blue-light absorbing proteorhodopsin precursor

ATGAATAAAATGTTACTGCCCTACGACATTGTAGGAGGATCTTTCTGGTTAATTTCTGTGGCAATGATCGGGGCAACACTCTTTTTCTTTTTTGAAAGAAGTAAAGTCAGCGTTCGTTGGCATACCCCCATGACAATGATCGCAGTAGTGTGTCTAATGTCATCTATTCACTACTTTTTTGTCAAAAATTTATGGGTTGTTAGTGGAACAGCACCCACACTTTTAAGATATATAGATTGGTTTTTAAATTTCCCTATGCAGGTACTTATATTTTACGCAATGTTGATGTCAGTAACCAAAGTAAAACAAGGAATGTTTTGGAGGTTGCTAGTCGGCACCTTGGTATTTATTACTGCAGAATTCTTAGGCGCTGCAGGCTATATGAGTAAAACTCTTGGTTTTATCGTTGGACTAGTTGGATGGCTTTACATATTAGGCGAGCTTTATGTTGGAGAAGCTGGTAGAGCAAATGCGAAATGTGGAAATGAAAATATTCAAATGGCTTTTTTCTCAAACCGACTAATACTTACAATAGGTTGGGCTATCTATCCAATGGGATATTTCATTGAACATTTAGGAGGTGGCATTGACCCAAACAGTGTAAACATAATATATAACCTTGCAGATTTCTTAAATAAAATTGTTTTTGGACTTATTATATACAAAGCAGCAACTCAAGATACGAGATCCAATGTTTAA

>marine_33279 Blue-light absorbing proteorhodopsin precursor

GGTGGAGTAGACGCTAACTCACTTAACGTGATTTACAACGCAGCTGACTTCTTGAACAAGATCGCTTTCGGTCTGATCATTTGGGCAGCAGCAATGCAACAACCTGGTAGAGCTAAGTAA

>marine_53208 Green-light absorbing proteorhodopsin precursor

ATGGAACTTACTTTTGGTCAATACGAACTGATCTACAACGCGTTTTCATTCGGTGTAGCGACGTTCTTCGCGGCGACCTTGTATTTCTGGCTGGGCCTTTCACAGGTCGCTAAAGAGTACAAGACTGCTGTCATCATCACTGGCCTCGTGACCTTCATCGCGGGTTACCACTACTGGCGTATCTTCGGTAGCTGGGGTGACGCGTATGAAGCAGTCAATGGCACTGTTACAGCCACTGGCGTTGCCTTCAACGTTGCCTATCGGTACGTTGACTGGTTGCTGACAGTACCCCTACTGCTGATCGAGTTGATCTTGGTCATGGGTCTGTCGAAATCAGAAACAGCCCGCAAGAGCATCACTTTGGGTGGCGCAGCTGCATTGATGATCATCTTAGGCTACCCTGGCGAAGTTTCAGGTGGTATCGATGGCACCCGCATGCTGTGGGGCGTCTTGTCGATGATTCCATTCCTGTACATCGTCTACAACCTGTTCGTTGGACTGTCTGATTCAATCAACAGCCAACCTTCAGAAGTACGTGGTTTGATCAGCACTGCACGTTGGATCGTGATCCTGTCATGGTCTTTCTACCCAGTCGTTTACTTCCTACCCTTCGCTATCCAAATGGACGGCGGTACGGCTAAGGCAGTCATCGAAGTGGGTTACACTGCAGCTGACATCATCGCTAAAGCAGCGTTTGGTGTATTGATCTTCACCATTGCTGTACGTAAATCACGCGGTGCCGCATAA

>marine_53716 Green-light absorbing proteorhodopsin precursor

ATGACCCTGAAATGGCTAAATAAGCTAGTGCCGGTAGGCATGCTAGCGACATTGTCATCAACGGCGTCAGCAGCTGCAAATTTGCAGTCAGATGACTTCGTTGGTATCTCGTTTTGGCTAATCTCGATGGCCCTTATGGCCTCAACGGTTTTCTTTTTATGGGAAACCCAATCCGTAAGTGGTAAGTGGAAAACGTCTCTTGTTGTGTCCGCGTTGGTTACCTTTATAGCGGCTGTCCATTATTTCTATATGCGTGATGTATGGATCGCGACAGGCGAAACCCCAACAGTGTATCGATACATTGACTGGTTACTCACTGTACCTTTATTGATGATCGAGTTCTTCTTGATTTTGCGTGCAATTGGTAATCCATCGGGCGGTATTTTCTGGCGCTTACTCGTCGGTACCTTGGTCATGTTGGTACCTGGTTTCATGGGTGAAGCAGGTTATATGGATGTAACAGTTGGATTTGTTATTGGTATGTTGGGTTGGTTCTACATCCTCTACGAAATCTTTGCAGGTGAGGCAGGCAAGATGGCAGCTGATAAAGCATCACCGGCTGTTCAGAGTGCTTACTCAACAATGCGTTGGATCGTAACTATCGGCTGGGCTATTTACCCACTGGGTTACGTGTTGGGTTACTTCGTTTACGTTGATGATGCTGGCGCAACAACACAAGCAGCCAGTGAAGCCCTGAATATTGTCTACAACCTAGCTGACGTTGTTAACAAGATCGCATTTGGTCTGTTGATTTGGTACGCAGCAACAGCAAGCAGCAAGGATGAGCAGCCTGCTTAA

>marine_119668 Green-light absorbing proteorhodopsin precursor

ATGTTCAATAAAAGTGTAAACGTGTGGCACAAAGCAGGGGTAGTCGGCCTGTTTGCGCTCGGTTCAAGCTCTGTGTTTGCAAGCACAATGCTTCAATCGGGTGACTTCGTGGGCTACTCATTCTGGTTAATATCTATGGCCTTGTCAGCCTCTACAATATTCTTCCTAATGGAAGCGTTACGTATGGGTGGCAAGTGGGCGACGAGTCTAACGGTATCGGCTTTGGTAACGTTCATCGCAGCCTTCCACTACATGTACATGCGTGAAGTGTGGGTGAGCACTGGAACGTCGCCAACGGATTTCCGTTACATCGACTGGTTGCTCACCGTGCCGCTGTTGATGATCGAGTTCTACTTGATCTTAGCTGCGATTACGAAGGTGTCTGGTGGTATCTTCTGGCGCCTGTTGATCGGTACCTTAGTAATGCTGGTCCCAGGTTACATGGGCGAGGCGGGTTACTTGAACGTCACAGTAGGCTTTGTAATCGGAATGCTAGGGTGGTTCTACATTCTGTACGAAATCTTTGCAGGCGAAGCAAGCAAGGTTGCCGCAGCGGAAGCCTCTCCGGCAGTACAAAAAGCGTACGGTTTGATGAAGTGGACAGTGACAATCGGCTGGGCAATTTACCCCATCGGTTACTTCTTGGGCTACATGGCCGGCGGGACGGATCAGGGTACTCTGAATATCGTGTACAACCTGGCAGACGTCCTGAACAAGATCGCGTTCGGCTTGTTCATTTGGTACGCAGCAAACGAAGACACTTCAGCGAAAGCGTAA
